# Evaluation of Psyllium (*Plantago ovata* L.) Husk Powder as a Stabilizer in Coconut Milk‐Based Probiotic Yogurt Production

**DOI:** 10.1002/fsn3.70085

**Published:** 2025-03-05

**Authors:** Ayca Gülhan, Hacer Çoklar, Mehmet Akbulut

**Affiliations:** ^1^ Department of Food Technology, Vocational School of Technical Sciences Aksaray University Aksaray Turkey; ^2^ Department of Food Engineering, Faculty of Agriculture Selcuk University Konya Turkey

**Keywords:** coconut milk, plant‐based yogurt, probiotic, psyllium husk, stabilizer

## Abstract

*Plantago ovata* L., also known as psyllium, is a plant native to the Mediterranean that is commercially cultivated and utilized for its polysaccharides, which have an arabinoxylan structure. Psyllium seeds possess various functional characteristics due to strong hydrophilic and gelling properties, as well as stabilizing and emulsifying capabilities. This study evaluated the use of psyllium ( *P. ovata* L.) husk powder (PHP) as a stabilizer in the production of probiotic plant‐based yogurt from coconut milk. The physicochemical, microbiological, textural, rheological, microstructural, and sensory properties of yogurt samples produced by adding different amounts of PHP (control, 0.125%, 0.25%, and 0.5%) during the storage period were analyzed. The produced plant‐based yogurts contained 72.80%–76.03% moisture, 18.30%–18.89% fat, 1.81%–2.03% protein, and 0.41%–0.44% ash. As the proportion of PHP and storage time increased, pH, and syneresis generally decreased and titratable acidity increased. The sample containing 0.5% PHP exhibited the highest microbiological counts. The addition of PHP to samples decreased L*, b*, C*, and *h* values and increased a* values. As the proportion of PHP increased, a significant increase in particle size parameters was detected. A more stable, homogeneous, and dense gel structure appeared in products with 0.25% PHP addition. The addition of PHP decreased hardness and adhesiveness while increasing cohesiveness. All samples exhibited a weak viscoelastic gel property (G′>G″). As the proportion of added PHP increased, the storage modulus decreased. Samples containing 0.25% PHP were rated higher in terms of sensory attributes, including taste, texture, and overall acceptability. Hierarchical cluster and principal component analyses were employed to categorize the yogurts, revealing that the control sample exhibited distinct characteristics compared to the psyllium‐added yogurts in terms of the aforementioned properties. The results show that, when added in the right amounts, PHP can stabilize the production of probiotic yogurt‐like products from coconut milk.

## Graphical abstract

Psyllium husk powder (PHP) was used as a stabilizer in the production of coconut milk‐based yogurt. PHP improved the textural, rheological, and microstructural properties of plant‐based yogurt. PHP contributed greatly to bacterial growth during fermentation. Adding 0.25% PHP was found to be appropriate in the production of coconut milk‐based yogurt.

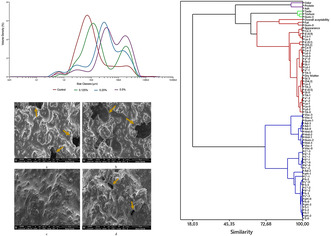

## 1 Introduction

In recent years, consumers have become increasingly interested in the positive effects of plant‐based dairy products on the environment and health. In addition, the increasing number of individuals with health problems such as lactose intolerance and allergies to milk proteins is increasing the demand for plant‐based dairy products day by day (Hickisch et al. 2016; Grasso et al. 2020). Plant‐based yogurts are usually made by fermenting aqueous extracts from different raw materials (legumes, oilseeds, grains, or nuts) (Grasso et al. 2020). The nutritional values of products made with plant‐based milks as alternatives to animal‐based milks are influenced by the plant source, processing, and fortification methods, as well as other added ingredients (Naibaho et al. 2022). Coconut milk can be used as a substitute for cow's milk as it has fewer calories, is rich in antioxidants, and contains certain vitamins such as folate, vitamin E, and minerals such as iron, calcium, potassium, magnesium, and zinc, as well as some other biomolecules (amino acids) (Tulashie et al. 2022). Studies have shown that yogurt alternatives can be produced from coconut milk with probiotic microorganisms and can maintain adequate probiotic levels during storage (Grasso et al. 2020; Boeck et al. 2021; Rasika et al. 2021; Naibaho et al. 2022). Different quality criteria, such as sensory, textural, rheological, and microstructural properties of yogurt, vary depending on various factors, such as fermentation procedure, milk and starter culture type, packaging process, and storage conditions (Choobari et al. 2021). Proteins, polysaccharides, and lipids form the complex gel system of yogurt (Fan et al. 2022). The biggest challenges faced by plant‐based yogurt manufacturers relate to the appearance and texture characteristics often caused by phase separation in such products. To simplify such plant‐based systems, the stabilization of proteins results in the formation of a discontinuous, weak gel, resulting in serum separation (Grasso et al. 2020). Various stabilizers, also known as hydrocolloids, are used to increase the consistency and viscosity of yogurt, reduce serum separation, and therefore provide stability to the lactic acid gel (Abdelmoneim et al. 2016). Stabilizers such as starch, alginate, carrageenan, methylcellulose derivatives, carob gum, and pectin are used in plant‐based yogurt production to reduce syneresis and improve the texture of yogurt (Fan et al. 2022). The *Plantago* genus belongs to the Plantaginaceae family and includes more than 200 species. *P. ovata* , *P. psyllium* and *P. indica* are the three important species of this genus. *P. ovata* L. plant is native to the Mediterranean region (Ladjevardi et al. 2015; Nazario Franco et al. 2020). Psyllium is the common name for the seeds of several members of the Plantago plant genus that are used commercially for mucilage production (Nazario Franco et al. 2020). *Plantago ovata* is a short‐stemmed, annual plant that grows between 30 and 40 cm and has many flowering shoots emerging from the base of the plant (AL‐Ogaidi and Alshaikh Daher 2019). Since ancient times, people have used psyllium as a medicinal agent around the world. They contain considered bioactive polysaccharides used in the treatment of many disorders and diseases, such as constipation, diarrhea, irritable bowel syndrome, inflammatory bowel disease (ulcerative colitis), colon cancer, diabetes, hypercholesterolemia, and high blood pressure (Madgulkar et al. 2014; Amini et al. 2018; Nazario Franco et al. 2020). Additionally, studies have shown that psyllium, a prebiotic ingredient, significantly boosts the growth of beneficial microorganisms in the digestive tract (Amini et al. 2018; Choobari et al. 2021). The main component of psyllium seeds is a mucilaginous polysaccharide (Madgulkar et al. 2014). The structure of gel‐forming mucilage consists of a highly branched arabinoxylan (Amini et al. 2018). The main structural units of arabinoxylan are β (1–4) linked arabinose and xylose (AL‐Ogaidi and Alshaikh Daher 2019). Psyllium seeds, a well‐known source for hydrocolloid production, have a functional role in foods due to their strong hydrophilic and gelling properties, as well as stabilizing and emulsifying capacity (Choobari et al. 2021; Nazario Franco et al. 2020). Studies have shown that psyllium gives successful results when used in yogurts produced from cow's milk due to its functional properties, such as its ability to form gel thanks to its high water‐holding capacity (Ladjevardi et al. 2015; Choobari et al. 2021). However, there are no studies on its use in yogurts made with plant‐based milk.

In this study, the use of psyllium husk powder (PHP) as a stabilizer to strengthen the network structure of yogurt gels produced with coconut milk was evaluated as a natural alternative to the use of other gums and hydrocolloids, which are considered additives. The physicochemical (pH, syneresis, titratable acidity, and color), microbiological, rheological, textural, microstructural, and sensory properties of coconut milk‐based probiotic yogurts were examined during a 21‐day storage period at 4°C.

## 2 Materials and Methods

### 2.1 Raw Materials

The organic coconut milk used in the study was obtained from Güzel Ada Food (Istanbul). In the production of plant‐based yogurt, Preoyogurt, one of the products of Preobio Healthy Living Products (Istanbul), was used as a yogurt starter. The yogurt starter contained *Lactobacillus delbrueckii* subsp. *bulgaricus*, *Streptococcus thermophilus* , *Lactobacillus acidophilus* strains, and inulin. Also, psyllium husk powder (PHP) from Güzel Ada Food (Istanbul) and sugar (Torku, Turkey) were obtained from a local supermarket. The label stated that 100 mL of coconut milk contained 17 g of fat, 1.8 g of carbohydrates, 1.8 g of protein, 2.5 mg of calcium, and 0.8 mg of iron. Yogurt starter culture contained *Lb. delbrueckii* subsp. *bulgaricus* (1 × 10^9^ CFU/g), *S. thermophilus* (3 × 10^9^ CFU/g), and *Lb. acidophilus* (1 × 10^9^ CFU/g). Also, 100 g of PHP contains 1 g of fat, 89.3 g of carbohydrates, 0.9 g of protein, and 0.17 g of salt.

### 2.2 Preparation of Coconut Milk‐Based Probiotic Yogurt

As a result of preliminary studies, the formulation of probiotic coconut milk‐based yogurts was determined. PHP was added to the coconut milk at rates of 0.125%, 0.25%, and 0.5% and mixed until it became homogeneous. The sample without added PHP was used as a control. All samples were supplemented with the addition of 5% sucrose as a lactose substitute so that the added starter cultures could show activity during fermentation. The coconut milk mixtures prepared in four different formulations were pasteurized at 90°C for 3 min. It was then cooled to 43°C. First of all, 2 g of lyophilized mixed yogurt culture were increased by activation. For the pre‐activation process, pasteurized and cooled to 43°C coconut milk was used. After completing the pre‐activation process, 4 g of active probiotic starter culture and coconut milk mixture were inoculated into the final plant milk volume of 600 mL of milk under aseptic conditions. The incubation at 43°C ± 1°C was terminated when the pH value of the samples reached 4.6 (Choobari et al. 2021; Pachekrepapol et al. 2021). The products obtained after fermentation were stored at 4°C for 21 days. Dry matter, protein, fat, ash analyses, and microstructural and sensory evaluations of coconut yogurts were performed after 24 h of storage at 4°C. Microbiological analysis, syneresis, pH, titratable acidity, rheological and textural properties, and color values were analyzed in the samples taken on the 1st, 7th, 14th, and 21st days of storage.

### 2.3 Physicochemical Analyses

#### 2.3.1 Total Dry Matter

Five grams of the prepared yogurt samples were weighed into the drying container and dried in the oven at 103°C ± 2°C until they reached a constant weight. Calculated results are expressed as percentages (AOAC 2005).

#### 2.3.2 Ash

The samples were incinerated in a muffle furnace at approximately 500°C until they turned into white ash. The ash content of the samples has been expressed as a percentage by weight (Pachekrepapol et al. 2021).

#### 2.3.3 Amount of Protein

The total protein amount was determined by the Kjeldahl method. Approximately 5 g of yogurt was weighed into the combustion tube. Ten grams of catalyst and approximately 13 mL of concentrated H_2_SO_4_ were added and mixed with the sample. The tubes were heated in the incinerator until a clear green color appeared. After the mixture in the sample was cooled, the distillation process was started. Then, the collected distillate was titrated with 0.1 N HCl, and the amount spent was determined. The protein ratio was determined by multiplying the calculated value by a factor of 6.38 (IDF 2013).

#### 2.3.4 Total Fat Analysis

Fat content of the samples was determined using the Gerber centrifuge device according to the Gerber method. First, 10 mL of sulfuric acid 95.0%–97.0% (*d*_25_ = 1.840 g/mL), then 11 mL of yogurt diluted in a 1:1 ratio, and finally 1 mL of amyl alcohol were added to the butyrometer and shaken slowly. Once the non‐oil elements were dissolved, the butyrometers were placed in a 65°C water bath for 5 min. Then, the butyrometers were centrifuged in the Gerber centrifuge device at 65°C for 5 min. The value read on the butyrometer scale is multiplied by 2 and given as the fat rate (IDF 2013).

#### 2.3.5 Determination of pH and Titratable Acidity (TA)

pH changes were determined on Days 1, 7, 14, and 21 using a Crison GLP 21 pH‐meter (Crison Instruments SA, South Africa) calibrated with appropriate buffer solutions (Aydoğan‐Coşkun et al. 2022). For TA analysis, 10 g of sample was taken, 10 mL of distilled water at 40°C was added, and the samples were titrated with 0.1 N NaOH until the pH value was 8.1. TA was calculated from the amount of NaOH consumed, and the results are given as “g lactic acid/100 g yogurt” (Coklar and Akbulut 2010).

#### 2.3.6 Determination of CIE Lab Color Parameters (L*, a*, b*, C* and *h*)

L*, a*, b*, C*, and *h* values of coconut milk yogurt samples were measured with a Konika Minolta CM‐5 model colorimeter (Konika‐Minolta, Osaka, Japan) equipped with a target mask that had an aperture of 30 mm diameter in a quartz petri dish. The petri dish (CM‐A128) with an inner diameter of 5.5 cm and a height of 3.5 cm was filled with yogurt to a height of 3.0 cm, and the color parameters of the product were measured. As color parameters, L* indicates brightness (L* = 0, black; L* = 100, white), +a* indicates redness, −a* indicates greenness, +b* indicates yellowness, and ‐b* indicates blueness. The *h* (hue) value shows the angle value in the color scale, while the C* value (chroma) shows the color saturation (Gülhan et al. 2023).

#### 2.3.7 Determination of Syneresis

To determine the rate of syneresis (%), 10 g of each sample was centrifuged at 4500 rpm for 20 min at 4°C. The separated supernatant was weighed, and the result was calculated using the following equation: (Devnani et al. 2020)

$$\text{Syneresis}\;\left(\%\right)=\frac{\text{Weight of supernatant}\;\left(g\right)\times 100\;}{\text{Weight of example}\;\left(g\right)}.$$

### 2.4 Steady and Dynamic Shear Rheology

The rheological measurements were carried out on an Anton Paar MCR 302 rheometer (Anton Paar, Autria) equipped with plate‐plate geometry (50 mm of diameter and a 1 mm gap). The temperature was fixed at 25°C using a Peltier system. The shear stress was measured at shear rates ranging from 0.01 to 100 s^−1^. The linear viscoelastic region (LVR) was determined through a strain sweep test at 10 rad/s angular frequency, and the viscoelastic properties of samples were carried out within the linear viscoelastic region. Frequency sweep measurements were made within the LVR range, over a frequency range of 0.01–100 rad s^−1^ (Lee and Lucey 2006).

### 2.5 Texture Profile Analysis

Texture analysis of coconut yogurt samples was carried out with a 50 kg load cell TA. XT Plus Texture analyzer (Stable Microsystems, Godalming, Surrey, UK). Measurement parameters: For 12 mm penetration, the test speed was 0.5 mm/s, and the speed before and after the test was set to 0.5 mm/s. A 10 mm cylindrical probe was used in the measurement, which was carried out with two consecutive compressions and was conducted at room temperature on samples stored at 4°C. Data were analyzed with Exponent software (Stable Microsystems) (Siefarth et al. 2014).

### 2.6 Microbiological Analyses

#### 2.6.1 Enumeration of *S. thermophilus*

To determine the number of *S. thermophilus* , cultivation was done according to the pour plate method using M17 agar medium. The number of *S. thermophilus* per gram was determined by counting the colonies (30–300) formed after incubation in petri dishes that were left for aerobic incubation for 48 h at 37°C (Bernat et al. 2015).

#### 2.6.2 Enumeration of *Lb. delbrueckii* subsp. *bulgaricus*

MRS agar, pH adjusted to 5.2 with 1.0 M HCl, was used for *Lb. bulgaricus* enumeration according to the pour plate method. The number of *Lb. bulgaricus* per gram was determined by counting the colonies (30–300) formed after anaerobic incubation for 72 h at 45°C (Ashraf and Smith 2015).

#### 2.6.3 Enumeration of *Lb. acidophilus*

Bile salts were added to MRS agar at a concentration of 1.5 g per liter to create a selective medium for counting *Lb. acidophilus* according to the pour plate method. The plates were incubated anaerobically at 37°C for 3 days. After incubation, colonies were counted. All microbiological results are given in log CFU/g (Choobari et al. 2021).

### 2.7 Scanning Electron Microscope (SEM) Analysis

The coconut yogurt samples stored at 4°C were first frozen at −80°C for 48 h and then lyophilized in a freeze dryer (Labogene ScanVac Coolsafe110–4, Lynge, Denmark) set at −110°C. The lyophilized samples were ground into powder and used for SEM analysis. The surface morphologies of the powder samples were examined using the FEI brand Quanta FEG 250 model SEM device. Prior to analysis, the samples were coated with gold–palladium using a Cressington sputter coater model 108 Auto from TED PELLA, INC. Images of the samples were acquired at a voltage of 10 kV and different magnifications using an EDAX‐Octane Pro system (Bilican 2023).

### 2.8 Size Distribution

Coconut yogurt samples stored at 4°C for 24 h were dispersed in 10 mL of distilled water at room temperature. Following dispersion, these samples were transferred to the dispersion unit of a Malvern brand MasterSizer‐3000 model particle size measurement device, which contained 500 mL of distilled water. Measurements were conducted using a 633 nm red laser and a 470 nm LED light source, with each measurement programmed to last for a minimum of 10 s for both light sources. The measurements included at least three parallel readings for each light source. The refractive index values of the samples and the distilled water used as the dispersant are 1.510 and 1.330, respectively. The analysis data, evaluated by the device software based on the Mie light scattering model, have been presented in the form of a distribution graph. The particle size parameters extracted from the software include the volume‐weighted mean (*D*_4,3_), the surface average diameter (*D*_3,2_), the particle diameter where < 10% of the gel particles are smaller (*d*(0.1)), the particle diameter where 50% of the gel particles are smaller (*d*(0.5)), and the particle diameter where < 90% of the gel particles are smaller (*d*(0.9)) (ISO 2020).

### 2.9 Sensory Analysis

The sensory properties of coconut milk yogurt samples were assessed by 20 untrained panelists (10 men and 10 women, 18–35 years old) who consumed yogurt frequently. Four different plant‐based yogurt samples were presented to the panelists after being stored at 4°C for 24 h. Panelists were asked to rate the appearance, taste, odor, texture, and overall acceptability of the yogurts using a 9‐point hedonic scale (1 = extreme bad and 9 = most perfect) (Choobari et al. 2021).

### 2.10 Statistical Analysis

Minitab version 21.3 software was used in the statistical analysis of the data obtained. The results are presented as means (SEM) from three independent experiments (*n* ≥ 3). Variability between the means of the results was determined by ANOVA and Tukey's multiplex analysis of variance. Significance levels were considered statistically significant at *p* ≤ 0.05. Principal component (PCA) and hierarchical cluster (HCA) analyses were also applied to the data for pattern recognition, and the samples were classified according to their similarities.

## 3 Results and Discussion

### 3.1 Total Dry Matter and Main Components (Protein, Fat, and Ash) of Coconut Milk‐Based Probiotic Yogurts

Table 1 provides the nutritional content of probiotic coconut milk yogurts made by adding PHP at various rates. The control sample made with coconut milk stored at 4°C for 1 day was found to contain 27.61% dry matter, 18.30% fat, 1.81% protein, and 0.41% ash. As the amount of PHP added to production increased, the dry matter value increased significantly (*p* < 0.05). Statistically significant changes were observed in the fat and protein contents of the samples (*p* < 0.05). According to the findings obtained from this study, it was understood that the ash amounts of all prepared samples varied between 0.41%–0.44% and had close values (Table 1). Grasso et al. (2020) reported that commercial coconut milk yogurt contained 4.90% fat, 4.20% saturated fat, 8.0% carbohydrate, and 0.60% protein. The protein and fat levels obtained from our study appear to be higher than those in the study of Grasso et al. (2020) (Table 1). Coconut milk‐based yogurt prepared by adding 1.0% tapioca starch contained 71.31% moisture, 1.91% protein, 6.01% carbohydrates, 20.22% fat, and 0.55% ash (Pachekrepapol et al. 2021). It appears that the nutritional content found in the current study is similar to the results of this research. According to a study (Craig and Brothers 2021) evaluating the nutritional contents from the nutrition label on the commercial packaging of coconut milk‐based yogurts sold in supermarkets in the western United States, they contained 5.5%–12.5% total fat, 5.0%–11.5% saturated fat, 10.0%–22.0% carbohydrates, and 0.3%–1.5% protein.

**TABLE 1:** Nutritional composition of probiotic coconut milk yogurts stored for 24 h at 4°C.

PHP (%)	DM (%)	Fat (%)	Protein (%)	Ash (%)
Control	27.61 ± 0.01^d^	18.30 ± 0.06^d^	1.81 ± 0.06^d^	0.41 ± 0.03^c^
0.125	28.16 ± 0.02^c^	18.38 ± 0.11^c^	1.88 ± 0.08^c^	0.44 ± 0.01^a^
0.25	28.40 ± 0.01^b^	18.89 ± 0.05^a^	2.03 ± 0.05^a^	0.41 ± 0.00^c^
0.5	29.20 ± 0.01^a^	18.55 ± 0.09^b^	1.94 ± 0.10^b^	0.42 ± 0.02^b^

*Note:* The difference between values marked with different letters in the same column is statistically significant (*p* < 0.05).

Abbreviation: DM, dry matter.

It was reported that a coconut milk‐based vegan rice pudding sample containing sugar and rice flour contained 3.29% oil, 0.73% protein, 0.35% ash, and 14.91% dry matter (Karimidastjerd et al. 2024). In pudding samples prepared with buffalo milk, 16% sugar, and 2% gelatin, coconut milk was added at 5%, 10%, and 15% levels instead of buffalo milk. As the proportion of coconut milk in the samples increased, so did the fat, total solids, protein, and carbohydrate content. While the fat content of puddings prepared with only buffalo milk was 8.50%, this value increased to 13.37% in the sample with 15% coconut milk added (Dawane et al. 2010). It is thought that the variety of raw materials and production methods used in the production processes may cause differences in the nutritional content of plant‐based yogurts.

### 3.2 pH and Titratable Acidity (TA)

The pH value of the control sample was determined to be 5.30 after 8 h of fermentation. The pH values of the samples with 0.125%, 0.25%, and 0.5% PHP added were 5.16, 5.16, and 5.05, respectively. At the end of 16 h of fermentation, the pH value of the control sample dropped to 4.62. The pH values of the samples with 0.125%, 0.25%, and 0.5% PHP added were 4.62, 4.60, and 4.57, respectively. The pH values of all samples have decreased with increasing storage time (Table 2). For the control samples stored at 4°C for 24 h, the pH value was 4.60, whereas at the end of the 21st day of storage, this value decreased to 4.45. Furthermore, as the ratio of added PHP increased, the pH values of the samples decreased. Throughout the 14‐day storage period, the decreases in pH of all samples were found to be statistically significant (*p* < 0.05). Similar to this, studies on plant‐based yogurts have shown a decrease in pH values during storage (Bernat et al. 2015; Pachekrepapol et al. 2021). Zhao et al. (2021) stated that the pH values of the yogurts prepared with almond milk varied between 4.4 and 4.6 after fermentation, and the pH value of almond yogurts decreased continuously during the cold storage period (21 days). Pachekrepapol et al. (2021) stated that the pH values of coconut milk‐based yogurts with tapioca starch decreased during the 14‐day storage period at +4°C and the pH values of the samples ranged from 4.49 to 4.58. It was understood that the pH results obtained in our study were consistent with the above‐mentioned studies. In the study examining the effects of a combination of polymerized whey protein, pectin, and xanthan gum on almond yogurt, pH values (4.26–4.35) were found to be lower than the results obtained in our study. It has been noted that *Lb. bulgaricus* continuously produces lactic acid, which is one of the reasons for the decrease in pH during storage (Shi et al. 2020).

**TABLE 2:** Changes in pH, TA, and syneresis values of probiotic yogurts based on coconut milk during 21 days of storage at 4°C.

PHP (%)	Storage (day)	pH	TA (%)	Syneresis (%)
Control	1	4.60 ± 0.01^aA^	0.33 ± 0.09^cC^	26.67 ± 0.30^aA^
	7	4.50 ± 0.00^aB^	0.37 ± 0.00^cB^	19.55 ± 0.58^aB^
	14	4.46 ± 0.01^aC^	0.40 ± 0.00^cA^	17.50 ± 0.74^aC^
	21	4.45 ± 0.04^aC^	0.39 ± 0.03^cA^	16.32 ± 0.49^aC^
0.125	1	4.58 ± 0.02^bA^	0.32 ± 0.02^cC^	23.31 ± 0.40^bA^
	7	4.48 ± 0.03^bB^	0.39 ± 0.01^cB^	17.40 ± 0.41^aB^
	14	4.44 ± 0.00^bC^	0.44 ± 0.04^bA^	16.12 ± 0.83^aB^
	21	4.42 ± 0.05^bC^	0.45 ± 0.08^bA^	15.79 ± 0.70^aB^
0.25	1	4.57 ± 0.00^bA^	0.35 ± 0.01^bC^	22.44 ± 0.54^bA^
	7	4.47 ± 0.01^cB^	0.41 ± 0.07^bB^	15.65 ± 0.69^bB^
	14	4.43 ± 0.03^bC^	0.44 ± 0.02^bA^	15.03 ± 0.88^aB^
	21	4.40 ± 0.02^bC^	0.47 ± 0.01^bA^	12.48 ± 0.56^bC^
0.5	1	4.55 ± 0.00^cA^	0.38 ± 0.05^aC^	17.84 ± 0.78^cA^
	7	4.47 ± 0.05^cB^	0.46 ± 0.07^aB^	13.42 ± 0.31^bB^
	14	4.40 ± 0.01^cC^	0.50 ± 0.01^aA^	11.24 ± 0.62^bB^
	21	4.37 ± 0.00^cC^	0.52 ± 0.00^aA^	11.01 ± 0.75^bB^

*Note:* There are significant differences in the mean values of different uppercase letters (A–D: storage time at 4°C) for products with the same formulation (*p* < 0.05). Similarly, the mean values of different lowercase letters (a–d: added PHP ratio) on the same storage day are significantly different (*p* < 0.05).

Lactic acid bacteria (LAB) play a crucial role in fermenting carbohydrates into lactic acid and other organic acids during yogurt production. The accumulation of acidic components in the environment contributes to the increase in milk acidity, which is also responsible for the coagulation process (Shori et al. 2022). It was observed that the TA values of yogurt samples with added PHP were higher than those of the control samples (Table 2), resulting in a statistically significant difference in the results (*p* < 0.05). An increase in TA values was observed with an increase in the ratio of PHP. Among the samples stored for 1 day, the highest TA value (0.38%) was observed in the product with 0.5% added PHP. On the 21st day of storage, this value increased significantly, reaching 0.52%. The increase in acidity values can be attributed to the fact that the prebiotic fibers in the composition of *P. ovata* L. stimulate the development of LAB. The TA values of probiotic yogurts have generally increased throughout the storage period, attributed to the continued metabolic activities of LAB during storage. The observed increase in TA values has been statistically significant (*p* < 0.05). In a study similar to ours, TA values rose significantly over 21 days of storage for cashew milk‐based yogurt samples that were added with different probiotics compared to the control (Shori et al. 2022). During the 28‐day storage period, the TA values of almond milk‐based yogurt samples increased, but the differences between the values from the 7th day were insignificant and detected around 2.2 g/L (Bernat et al. 2015). Commercial coconut yogurt has a TA of 0.49 mL NaOH/g and a pH of 4.00 (Grasso et al. 2020). The study determined the pH and TA values of commercially available plant‐based yogurt samples, finding the pH values of almond milk‐based samples to be between 3.99 and 4.01 and the TA values in terms of lactic acid to be between 0.52% and 0.70%. The soy yogurt sample in the same study had a pH value of 4.23 and a TA value of 0.79%. Yogurts produced from cow's milk have been found to have a pH value of 3.89 and a TA of 1.78%. This difference compared to plant milk‐based samples was attributed to the higher buffering capacity of cow's milk compared to almond and soy beverages (Devnani et al. 2020). We observe lower pH and higher TA values when comparing the findings of this study with existing research. Devnani et al. (2020) attribute this situation to the higher protein concentration (4.9% in bovine milk compared to < 1% in almond milk) and the naturally occurring salts and organic acids present in milks. In the study related to probiotic yogurts produced from pasteurized cow's milk containing 1% fat, it was indicated that samples containing *P. ovata* Forsk seed mucilage had a lower pH and higher TA compared to the control sample. Additionally, the study notes that the samples' pH decreases and their acidity increases as the storage period increases. *Plantago ovata* 's effect has caused the bacterial population to produce more lactic acid, which explains this situation (Choobari et al. 2021). The changes in pH and TA observed in the production of low‐fat yogurt gel using psyllium husk gum as a fat replacer in a study were consistent with the findings obtained from our study. The presence of highly branched acidic arabinoxylan in psyllium's structure, which allows bacteria like *S. thermophilus* and *Lb. delbrueckii* ssp. *bulgaricus* to survive in greater numbers in this food matrix, explains the higher acidity or lower pH in yogurts attributed to psyllium husk gum (Ladjevardi et al. 2015).

### 3.3 Syneresis

The syneresis value is the measure of serum released from yogurt gel subjected to centrifugal force (Pachekrepapol et al. 2021). The syneresis values of the produced probiotic yogurts during storage at 4°C are given in Table 2. Addition of PHP to coconut yogurts significantly reduced syneresis values, especially in samples stored for 1 day (*p* < 0.05). It was seen that as the amount of PHP added to the yogurt samples increased, the syneresis values decreased as a result of binding of free water. The syneresis values of all samples decreased during the 21‐day storage period. This decrease was found to be statistically different compared to the first day of storage (*p* < 0.05). In the samples stored at 4°C for 1 day, the lowest degree of syneresis was detected to be 17.84% in the sample produced by adding 0.5% PHP. In the same sample, this value decreased to 11.01% after 21 days of storage. In the control sample, the highest syneresis value (26.67%) was detected at the end of 1 day of storage. A similar change was previously reported in the coconut milk‐based yogurt with tapioca starch (Pachekrepapol et al. 2021). In the studies of other plant‐based yogurt alternatives, it was observed that chickpea, soybean, and lupin‐based products produced syneresis values at levels similar to those determined in our study (Hickisch et al. 2016; Wang et al. 2018). In almond milk‐based yogurt samples where no stabilizer is used, the serum‐holding capacity was reported at 43% on the first day of storage. This situation has been attributed to the formation of a weak gel structure (Bernat et al. 2015). Researchers have noted that adding thickeners and gelling agents like guar gum, pectin, natural, or modified starch lowers the syneresis levels in commercial yogurt‐like products based on plant milk (Grasso et al. 2020; Devnani et al. 2020). It has been stated that pectin, carob gum, and inulin are used as stabilizers and thickening agents in yogurt alternatives sold commercially in the USA (Craig and Brothers 2021). After 14 days of storage, syneresis was 50% lower in a study that added xanthan gum, pectin, and polymerized whey protein as stabilizers and gelling agents to almond milk‐based yogurts. It has been emphasized that decreases in the syneresis values of the almond yogurt enriched with polymerized whey protein, pectin, and xanthan gum at the end of 14 days of storage are due to the increase in the water‐holding capacity of the gel during storage (Shi et al. 2020). According to a study (Choobari et al. 2021) that added *P. ovata* Forsk seed mucilage at concentrations of 0.5%, 1%, and 2% to yogurt production from cow's milk, the syneresis value decreased as the concentration of *P. ovata* and storage time increased. Researchers have noted that adding *P. ovata* to yogurt samples increases osmotic activity and absorbs free water, which in turn reduces the syneresis of the samples. The highest syneresis values were achieved when psyllium ( *P. ovata* Forsk) hydrocolloid gel was added to liquid Kashk at a concentration of 0.1% (Amini et al. 2018). Different almond milk‐based yogurt samples prepared with varying amounts of protein, fat, and sugar added showed water‐holding capacity ranging from 27.66% to 52.22%. The higher the total solid content, the lower the risk of serum separation (Zhao et al. 2021). As the total solid content of the yogurt increased, the syneresis value decreased. However, the addition of psyllium, due to its high water‐binding capacity, resulted in lower syneresis values in yogurt samples. This indicates that PHP may act as a stabilizer in the production of plant‐based yogurt‐like products.

### 3.4 Microbiological Count Results of Coconut Milk‐Based Probiotic Yogurts

Coconut milk is one of the main carrier matrices used in the development of probiotic plant‐based foods (Rasika et al. 2021). Probiotics are defined as live microorganisms that, when consumed in adequate amounts, can perform beneficial functions for their hosts, as recognized by the FAO/WHO. Lactic acid bacteria (LAB) have many functions, such as keeping the balance of microbes in the digestive system, controlling endotoxins, inhibiting the growth of harmful bacteria, and making the body's immune system stronger (Fan et al. 2022). Fermented plant‐based milk products need to contain a minimum of 6–7 log CFU/g of live probiotic bacteria until the end of their shelf life to exhibit probiotic properties (Shori et al. 2022). In this study, it was found that the counts of *S. thermophilus* and *Lb. delbrueckii* subsp. *bulgaricus* in probiotic yogurt products containing PHP were above this value, while the count of *Lb. acidophilus* was between 6 and 7 log CFU/g up to the 14th day of storage (Table 3). Since the number of *Lb. acidophilus* decreased below 6 log cfu/g after the 14th day of storage, the product lost its probiotic properties. It has been emphasized that *P. ovata* Forsk seed mucilage contains certain chemical substances such as D‐xylose, arabinose, D‐galactose, D‐galacturonic acid, and fibers, which are believed to have prebiotic properties (Choobari et al. 2021). Due to its prebiotic effect, *P. ovata* has led to higher counts of *Lb. delbrueckii* subsp. *bulgaricus*, *S. thermophilus* , and *Lb. acidophilus* compared to the control sample. Similarly, Pandey et al. (2016), Firooz et al. (2019), and Choobari et al. (2021) have reported that *P. ovata* mucilage enhances the survival ability of probiotic bacteria. AL‐Ogaidi and Alshaikh Daher (2019) have mentioned that arabinoxylan oligosaccharides are polysaccharides found in the psyllium plant, which could potentially be new prebiotics similar to inulin and fructo‐oligosaccharides. Adding PHP usually caused a statistically significant rise (*p* < 0.05) in the numbers of *Lb. delbrueckii* subsp. *bulgaricus* and *S. thermophilus* as the concentration went up. Storage duration significantly influenced the LAB counts in probiotic yogurts (*p* < 0.05). Up to the 14th day of storage, the counts of *Lb. delbrueckii* subsp. *bulgaricus* and *S. thermophilus* went up in samples with 0.25% and 0.5% PHP. By Day 21 of storage, the counts had significantly decreased in all samples (*p* < 0.05). This decrease indicates that the growth conditions in the yogurts encouraged the development of LAB until Day 14, but subsequently, the increased acidity and metabolic products negatively affected their growth. The lowest count of *Lb. delbrueckii* subsp. *bulgaricus* was observed in the control sample on Day 21 of storage (6.62 log CFU/g), while the highest count was found in the sample with 0.5% PHP added on Day 14 of storage (8.47 log CFU/g). The counts of *S. thermophilus* in yogurt samples ranged from 7.41 to 8.66 log CFU/g during the 21‐day storage period. The sample prepared with 0.5% PHP addition showed the highest counts of *S. thermophilus* throughout the storage period. Generally, the samples showed a higher count of *S. thermophilus* than *Lb. delbrueckii* subsp. *bulgaricus* throughout the storage period. The yogurt prepared with different levels of tapioca starch added to coconut milk showed LAB counts ranging from 5.96 to 6.45 log CFU/g during the 14‐day storage period (Pachekrepapol et al. 2021). Examining the research findings reveals that our study maintained higher levels of probiotic bacteria throughout the storage period. In cashew yogurt made with *S. thermophilus* and *L. delbrueckii* subsp. *lactis*, *S. thermophilus* levels changed from 8.08 to 8.15 log CFU/mL over the 14‐day storage period. Like our study, they dropped significantly by Day 21 of storage (Shori et al. 2022). The count of *Lb. acidophilus* in yogurt samples ranged from 4.95 to 7.40 log CFU/g. The addition of 0.25% PHP had a statistically significant effect (*p* < 0.05) on the count of *Lb. acidophilus* in the yogurts. The counts of *Lb. acidophilus* in all samples increased by the 7th day of storage and decreased below the initial value by the end of storage. Similarly, Huang et al. (2022) reported a significant increase in the count of LAB in their quinoa‐based yogurt samples by the 7th day of storage, compared to samples stored for 1 day, followed by a decrease by Day 21. The *Lb. acidophilus* counts revealed that yogurt samples with *P. ovata* Forsk seed mucilage at varying concentrations (0.5%, 1%, and 2%) had more live bacteria than the control sample, and these samples showed less decrease over the storage period. On the first day of storage, the yogurt sample containing 2% *P. ovata* Forsk seed mucilage had the highest count of *Lb. acidophilus* (6.68 log CFU/g) (Choobari et al. 2021). We examined the microbiological count results throughout storage and found that *Lb. delbrueckii* subsp. *bulgaricus* and *S. thermophilus* were present in higher numbers in all samples compared to *Lb. acidophilus*. Similar to our current study, the population of *Lb. acidophilus* in the produced probiotic almond yogurt decreased rapidly as the storage period increased, starting at 10^6^ CFU/g in the first week. The accumulation of lactic acid and acetic acid produced by *Lb. delbrueckii* subsp. *bulgaricus* during storage led to a decrease in pH, which was the main reason for the loss of viability of *Lb. acidophilus* (Shi et al. 2020). *Plantago ovata* powder (%0.125 and %0.50) increased the logarithmic count of *L. rhamnosus* by 11.48% in a study that used it as a prebiotic in fermented milk production (AL‐Ogaidi and Alshaikh Daher 2019). Another study fermented almond milk with probiotic *L. reuteri* and *S. thermophilus* , resulting in a significant decrease in LAB during a 28‐day storage period. However, the LAB count remained at the recommended minimum level (10^7^ CFU/mL) throughout the storage period (Bernat et al. 2015). Samples were taken immediately after inoculation (Day 0) and after 30 days of storage at 4°C in a study investigating cashew milk as a matrix for providing probiotic bacteria. On Day 0, the *Lb. acidophilus* count was 8.17 log CFU/mL, and on Day 30, it was 8.89 log CFU/mL. On Day 0, the count of *L. plantarum* was 8.04 log CFU/mL, and on Day 30, it was 8.38 log CFU/mL. The pH of cashew milk decreased from 6.45 on Day 0 to 5.65 on Day 30 (Bruno et al. 2020). The fact that the pH value did not fall below 4.5 may be the reason for the more dense microbial population compared to our current study results.

**TABLE 3:** Microbiological count results of coconut milk‐based probiotic yogurts during 21 days of storage at 4°C.

PHP (%)	Storage (day)	*Lb. delbrueckii* subsp. *bulgaricus* (log CFU/g)	*S. thermophilus* (log CFU/g)	*Lb. acidophilus* (log CFU/g)
Control	1	7.18 ± 0.11^cC^	7.64 ± 0.08^dB^	6.45 ± 0.15^bA^
	7	7.37 ± 0.14^dA^	7.78 ± 0.11^dA^	6.62 ± 0.09^bA^
	14	7.31 ± 0.09^dB^	7.49 ± 0.05^dC^	6.01 ± 0.15^cB^
	21	6.62 ± 0.15^dD^	7.41 ± 0.13^dD^	4.95 ± 0.10^cC^
0.125	1	7.45 ± 0.18^bB^	7.73 ± 0.13^cC^	6.51 ± 0.10^bA^
	7	7.78 ± 0.12^cA^	8.14 ± 0.09^cA^	6.75 ± 0.13^bA^
	14	7.45 ± 0.11^cB^	8.07 ± 0.10^cB^	6.20 ± 0.14^bB^
	21	7.09 ± 0.16^cC^	7.57 ± 0.07^cD^	5.60 ± 0.11^bC^
0.25	1	7.79 ± 0.13^aB^	7.92 ± 0.17^bB^	6.88 ± 0.08^aB^
	7	8.09 ± 0.06^bA^	8.39 ± 0.08^bA^	7.15 ± 0.11^aA^
	14	8.12 ± 0.13^bA^	8.41 ± 0.19^bA^	6.24 ± 0.15^bC^
	21	7.72 ± 0.05^bC^	7.67 ± 0.09^bC^	5.62 ± 0.18^bD^
0.5	1	7.81 ± 0.08^aC^	8.41 ± 0.10^aB^	7.11 ± 0.06^aB^
	7	8.34 ± 0.17^aB^	8.65 ± 0.15^aA^	7.40 ± 0.17^aA^
	14	8.47 ± 0.08^aA^	8.66 ± 0.04^aA^	6.58 ± 0.09^aC^
	21	7.80 ± 0.11^aC^	7.94 ± 0.08^aC^	5.76 ± 0.13^aD^

*Note:* There are significant differences (*p* < 0.05) in the mean values of different capital letters (A–D: storage time at 4°C) in the product with the same formulation. The mean values of different lowercase letters (a–d: proportion of PHP added) on the same storage day are significantly different (*p* < 0.05).

### 3.5 Color Parameters

Table 4 displays the L*, a*, b*, C*, and *h* color values of coconut milk‐based yogurts measured during 21 days of storage at 4°C. The brightness (L*) of yogurts is correlated with the particle size of both fat globules and protein, which affects their ability to reflect and scatter light (Grasso et al. 2020). A low L* value indicates a brighter appearance of the food (Huang et al. 2022). On the first day of storage, as the proportion of added PHP increased in yogurt samples, L* values decreased (*p* < 0.05), with the control sample having a higher L* value compared to other yogurts. The control sample again had the highest L* value (90.32) on Day 21 of storage. In the control sample, there was a significant increase in L* values (*p* < 0.05) after the 7th day of storage, indicating a perception of a whiter color. Similarly, in other samples, statistically significant (*p* < 0.05) increases and decreases in L* values generally occurred as storage time increased. Similar to the changes observed in L* values of our study, an increase in the concentration of psyllium ( *P. ovata* Forsk) hydrocolloid gel included in the liquid Kashk formulation led to a significant decrease in the L* value (Amini et al. 2018). During Days 1, 7, 14, and 21 of storage, as the proportion of PHP increased in all samples, the a* value statistically significantly increased (*p* < 0.05). Yogurt samples containing 0.25% and 0.5% PHP showed increases after the 7th day of storage. On the first day of storage, the control sample had the highest b* value at 6.97, while the yogurt sample with 0.5% PHP had the lowest value at 6.13. As the PHP rate added during storage increased, statistically non‐significant decreases in b* values were observed (*p* > 0.05). However, the decreases observed in the sample with 0.5% PHP were statistically significant (*p* < 0.05). The evaluation of the L*, a*, and b* values of probiotic yogurts revealed that the sample containing 2% *P. ovata* Forsk seed mucilage had significantly lower L* and b* values compared to the control sample, while the a* value was higher (Choobari et al. 2021). In this study, the changes in color parameters were consistent with our research results. The addition of PHP also affected the C* and *h* values, and generally, as the proportion increased, decreases in C* and *h* values were observed (*p* < 0.05). Increases and decreases in C* and *h* values occurred with increasing storage time for each formulation. Yogurt samples produced by adding 0.5% PHP exhibited the lowest C* and h values. We observed decreases in brightness, color saturation, and yellowness values, along with an increase in redness values, as the proportion of PHP added to the yogurt samples increased. Changes due to the pigmentation of *P. ovata* affected the color parameters of the yogurts. Grasso et al. (2020) reported the L*, a*, and b* values of coconut yogurts as 62.3, −1.79, and 4.31, respectively. Yilmaz‐Ersan and Topcuoglu (2022) found that probiotic almond milk‐based yogurt samples went from having higher a* values to having lower *h* values over a 21‐day storage period. These changes are similar to what we saw in our study. In the study conducted by Zhao et al. (2021), the L*, a*, and b* values of almond milk‐based yogurt samples were reported as 81.61, 0.94, and 9.56, respectively. It was stated that there was a positive correlation between the L* and a* values of the products and the fat content (%), and a significant association between the b* values and the protein content (%). In the study conducted by Bernat et al. (2015), the brightness and yellowness values of yogurt produced from almond milk were reported to be lower than the values found by Zhao et al. (2021). Variations in the proteins, fats, and additives included in the formulation may account for this difference.

**TABLE 4:** Color parameters of coconut milk‐based probiotic yogurts during 21 days of storage at 4°C.

PHP (%)	Storage (day)	L*	a*	b*	C*	*h*
Control	1	89.88 ± 0.08^aB^	0.09 ± 0.03^dB^	6.97 ± 0.04^aA^	6.97 ± 0.04^aA^	89.25 ± 0.28^a^
	7	88.92 ± 0.18^bC^	0.11 ± 0.01^dA^	6.99 ± 0.07^aA^	6.99 ± 0.07^aA^	89.12 ± 0.07^a^
	14	89.54 ± 0.11^aB^	0.02 ± 0.00^dC^	6.84 ± 0.07^aB^	6.84 ± 0.07^aB^	89.44 ± 0.41^a^
	21	90.32 ± 0.05^aA^	0.10 ± 0.04^dB^	6.60 ± 0.02^aC^	6.60 ± 0.02^bC^	89.16 ± 0.34^a^
0.125	1	89.46 ± 0.07^aA^	0.39 ± 0.02^cA^	6.95 ± 0.01^aA^	6.96 ± 0.01^aA^	86.81 ± 0.11^bC^
	7	89.14 ± 0.48^aA^	0.31 ± 0.05^cB^	6.93 ± 0.15^aA^	6.93 ± 0.15^aA^	87.45 ± 0.37^bB^
	14	88.88 ± 0.08^bB^	0.17 ± 0.05^cC^	6.68 ± 0.06^aB^	6.68 ± 0.06^bB^	88.53 ± 0.43^bA^
	21	89.49 ± 0.15^bA^	0.31 ± 0.03^cB^	6.70 ± 0.03^aB^	6.71 ± 0.03^aB^	87.36 ± 0.24^bB^
0.25	1	88.39 ± 0.04^bB^	0.58 ± 0.02^bA^	6.90 ± 0.01^aA^	6.92 ± 0.01^aA^	85.24 ± 0.10^cB^
	7	88.42 ± 0.10^bB^	0.47 ± 0.02^bB^	6.79 ± 0.06^aB^	6.80 ± 0.06^bB^	86.06 ± 0.08^cA^
	14	89.45 ± 0.15^aA^	0.48 ± 0.05^bB^	6.39 ± 0.01^bC^	6.41 ± 0.01^cC^	85.76 ± 0.39^cB^
	21	88.36 ± 0.12^cB^	0.56 ± 0.00^bA^	6.73 ± 0.06^aB^	6.76 ± 0.06^aB^	85.23 ± 0.03^cB^
0.5	1	86.90 ± 0.02^cB^	0.73 ± 0.01^aB^	6.13 ± 0.04^bA^	6.17 ± 0.04^bB^	83.25 ± 0.03^dA^
	7	87.96 ± 0.17^cA^	0.70 ± 0.02^aC^	5.98 ± 0.03^bB^	6.02 ± 0.03^cC^	83.34 ± 0.14^dA^
	14	85.19 ± 0.44^cC^	0.74 ± 0.01^aB^	6.20 ± 0.14^bA^	6.24 ± 0.13^dA^	83.19 ± 0.08^dA^
	21	87.17 ± 0.01^dA^	0.78 ± 0.00^aA^	6.14 ± 0.02^bA^	6.19 ± 0.02^cB^	82.77 ± 0.05^dB^

*Note:* There are significant differences (*p* < 0.05) in the mean values of different capital letters (A–D: storage time at 4°C) in the product with the same formulation. The mean values of different lowercase letters (a–d: proportion of PHP added) on the same storage day are significantly different (*p* < 0.05).

### 3.6 Particle Size Analysis (Mastersizer)

**FIGURE 1:**
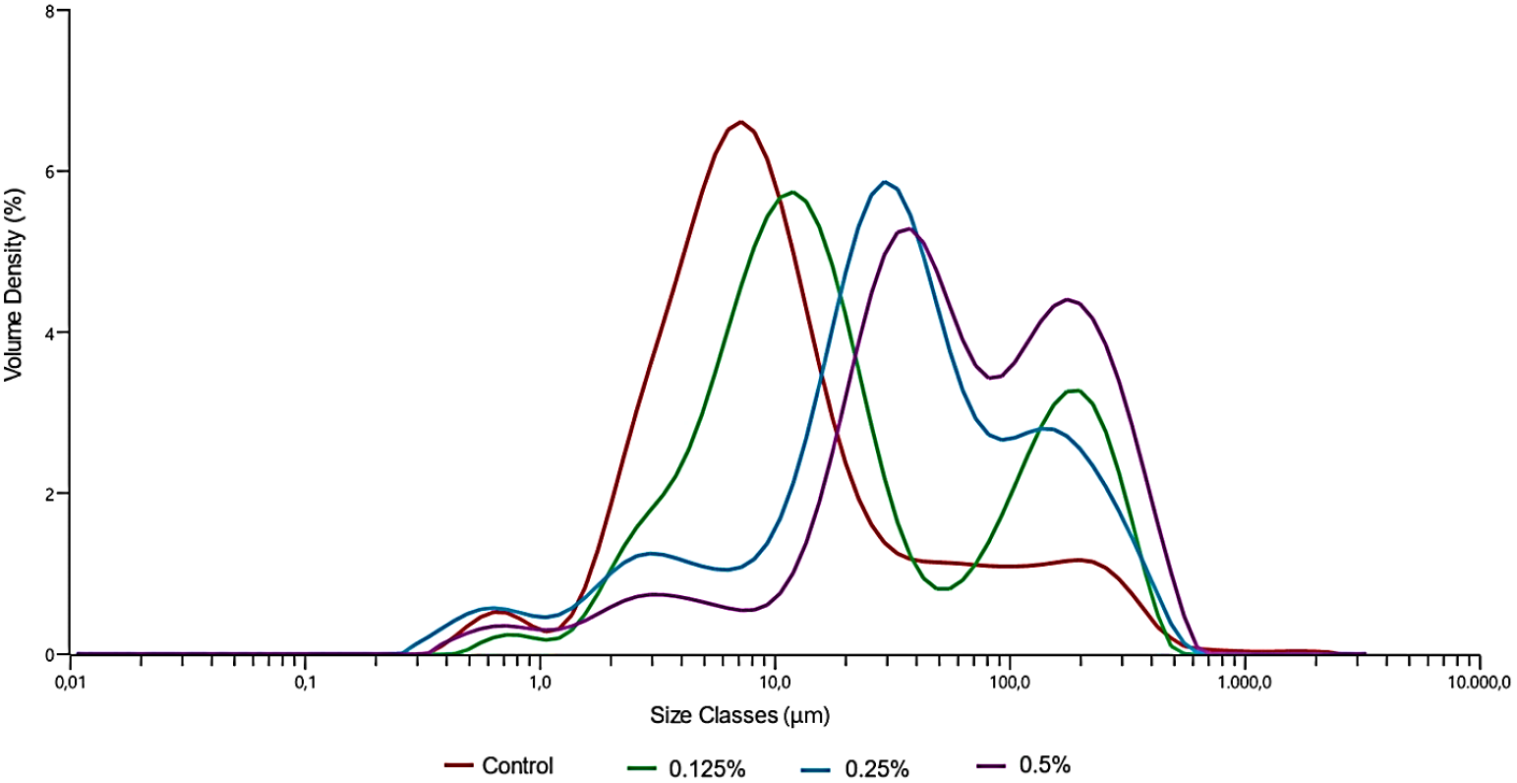
Particle size distribution of yogurt samples.

The intensity of light scattering strongly influences the particle size in colloidal systems, according to the Fraunhofer diffraction principle (Amini et al. 2018). The stability and texture of yogurt are primarily dependent on the particle size (Zhao et al. 2021). Figure 1 displays the particle size distribution of coconut milk‐based probiotic yogurts stored at 4°C for 1 day, while Table 5 provides the particle size parameters ((*D*_4,3_), (*D*_3,2_), *d*(0.1), *d*(0.5), and *d*(0.9)). Generally, as the ratio of PHP in coconut milk yogurts increases, a significant increase (*p* < 0.05) in the tested particle size values is observed. The control sample contained smaller particles compared to the other samples. As the ratio of PHP in coconut milk yogurts increases, the system's average particle size generally increases. The (*D*_4,3_) value increased from 37.1 μm in the control to 105 μm in the sample with 0.5% added PHP. Similarly, the (*D*_3,2_) value increased from 5.24 μm to 12.7 μm, the *d*(0.1) number increased from 2.59 μm to 8.92 μm, the *d*(0.5) number increased from 8.24 μm to 56.7 μm, and the *d*(0.9) number increased from 101 μm to 269 μm. PHP, known for its role in coagulation and gelling, significantly influences the values of particle size. This study used PHP as a stabilizing agent for gelation, and its effect on particle size likely varied depending on the added concentration. Because of the stabilizing effect, the second peak observed in samples with added PHP likely represents swollen granules, lipid‐protein clusters, and polysaccharides (such as pectin and fiber). It is known that particles larger than 0.1 μm in yogurt gels affect the stability, texture, and sensory properties of yogurt (Devnani et al. 2020). Psyllium hydrocolloid gel, when added in high amounts to liquid Kashk formulations, increases the unabsorbed anionic psyllium polysaccharides on casein micelles through the depletion flocculation phenomenon, leading to an increase in particle size in the system (Amini et al. 2018). According to Zhao et al. (2021), the particle size values ((*D*_4,3_), (*D*_3,2_), *d*(0.1), *d*(0.5), and *d*(0.9)) of yogurt samples produced with almond milk were determined to be 79.91 μm, 8.00 μm, 8.67 μm, 57.15 μm, and 171.94 μm, respectively. The study observed a significant increase in all particle size values as the protein and fat contents of the samples increased. The differences in particle size values obtained in the studies have been emphasized as being associated with the fat and protein content of the products. Researchers measured the particle sizes of plant‐based yogurts and found similar locations for the two peak points in three types of commercial almond yogurts. The first peak at 1 μm likely corresponds to weakly associated protein particles or aggregates that separate during dilution and mixing. The second peak at about 42 μm probably shows swollen starch granules and lipid‐protein clusters seen in confocal images. It also shows polysaccharides (like pectin, gums, and fibers) that are present in the matrix but are not colored. In general, the average particle size of almond milk‐based systems was found to be larger than soy and animal milk‐based systems. The size of all measured particles was below 150 μm. It was reported that the particle sizes below 150 μm did not adversely affect the sensory properties of yoghurts (Devnani et al. 2022).

**TABLE 5:** Particle size values (μm) of coconut milk‐based probiotic yogurts stored for 24 h at 4°C.

PHP (%)	(*D*_4,3_)	(*D*_3,2_)	*d* (0.1)	*d* (0.5)	*d* (0.9)
Control	37.1	5.24	2.59	8.24	101
0.125	61.2	8.81	3.83	15.1	206
0.25	67.9	7.26	3.15	32.6	191
0.5	105	12.7	8.92	56.7	269

### 3.7 Scanning Electron Microscope (SEM) Analysis

**FIGURE 2:**
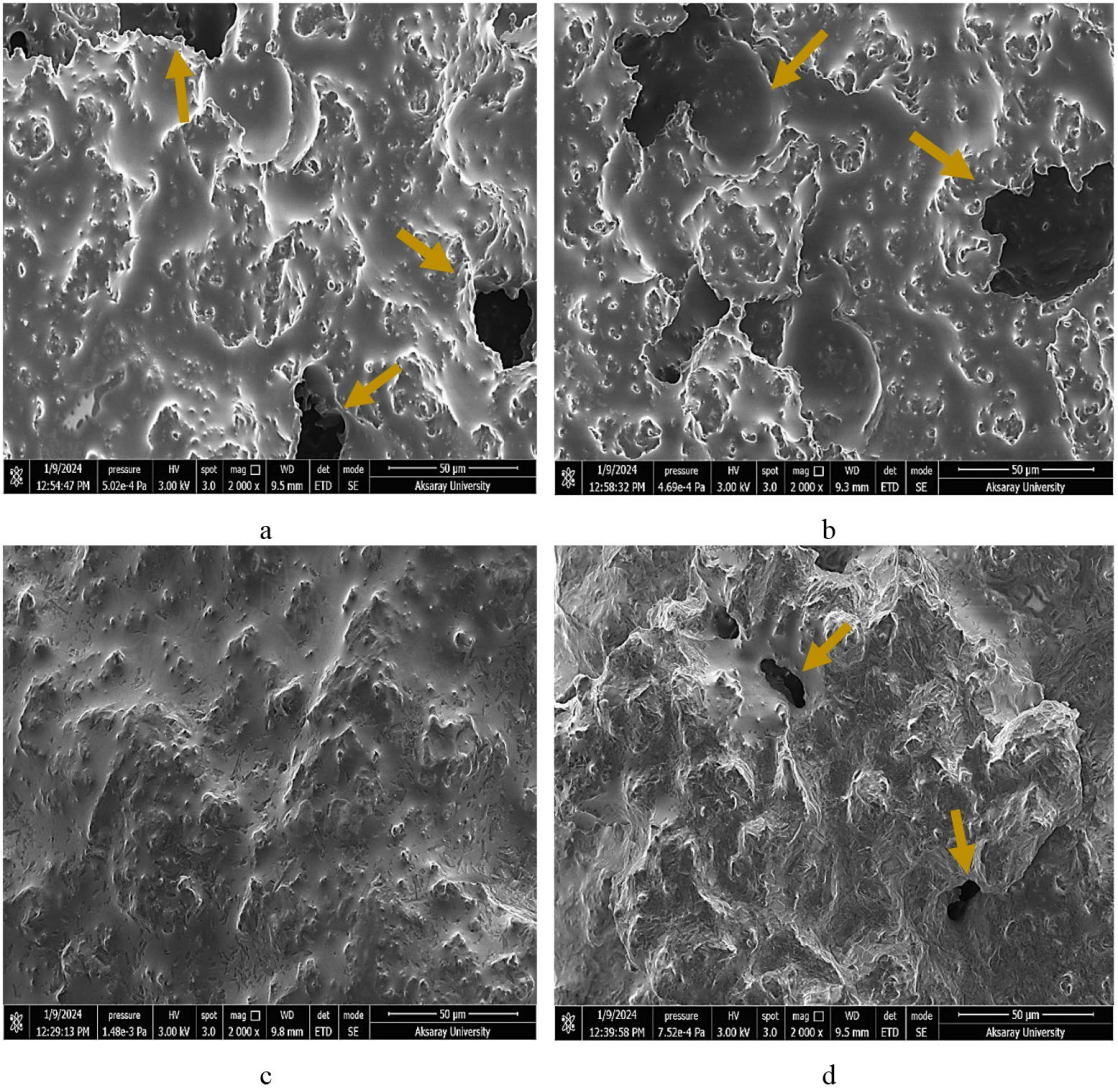
SEM image of a coconut milk‐based yogurt samples: (a) control, (b) 0.125% PHP, (c) 0.25% PHP, (d) 0.5% PHP. Solid arrow = voids.

SEM images revealed differences in gel structures based on PHP addition ratios in coconut yogurts (Figure 2a–d). The large voids indicated by the solid arrow were actually occupied by serum and fat. The control sample showed a structure with open and larger voids (a). The pore size became smaller, especially when 0.5% PHP was added to coconut milk (*d*). When 0.25% PHP was added to coconut milk, the network of coconut protein became more compact and denser (c). However, the sample (b) prepared by adding 0.125% PHP to coconut milk was looser compared to the samples added with 0.25% and 0.5% PHP. The control sample had a more varied microstructure in SEM images compared to the others. It had fat and protein aggregates of different sizes and shapes, as well as clumps of protein particles and lipid droplets. The addition of PHP significantly influenced the microstructure of coconut yogurt. The probiotic yogurts prepared with a 0.25% PHP showed the most homogeneous distribution. We observed a uniform gel structure with minimal porosity in this sample. This result is consistent with the highest score for the texture parameters in sensory analysis. Overall, the interconnected network of fat and protein formed the gel in the control sample, while *P. ovata* acted as a stabilizer in other samples, thereby affecting the structure. The PHP stabilizing effect, with a more homogeneous distribution and fewer aggregated pores, indicated that adding it at a 0.25% ratio was appropriate. Unlike the samples containing PHP, the control sample had a gel appearance and contained larger particles or aggregates. The control sample had a higher synergistic value, which meant it was less stable. This was in line with the fact that the sample was not uniform in appearance, with more clumped particle aggregates than a creamy gel‐like structure. The study examined the microstructure of almond milk yogurts and found that the control group, lacking any stabilizer, displayed a honeycomb‐like structure with large voids. The addition of whey protein concentrate to almond milk resulted in a decrease in pore size and a more compact and dense almond protein network. However, samples produced by adding a combination of whey protein concentrate, pectin, and xanthan gum showed a more homogeneous, uniform microstructure (Shi et al. 2020). Also, when a small amount (0.1%) of psyllium was added to the liquid Kashk mixture, SEM pictures showed that continuous amorphous aggregates of casein particles of different sizes were formed. However, adding psyllium at levels higher than 0.1% resulted in a smoother particle distribution and a stable structure (Amini et al. 2018). Additionally, yogurts stabilized with higher amounts of psyllium husk gum (0.10% and 0.12%), compared to those made with 0.08% psyllium husk gum, had a microstructure that was denser and less porous. The higher concentration of psyllium husk gum led to stronger intermolecular interactions between arabinoxylan chains and casein micelles (Ladjevardi et al. 2015). According to the images obtained from the confocal laser scanning microscope (CLSM), the size and shape of clusters in almond yogurt varied depending on the compositional variation. Protein supplementation up to 6% contributed to the formation of a more compact protein–protein matrix and ensured a more homogeneous distribution of fat globules within the protein matrix. As the fat content decreased from 7% to 0.8%, the size of fat droplets significantly decreased, and they were more uniformly trapped within the protein matrix (Zhao et al. 2021). The study using CLSM examined the microstructure of plant‐based yogurts and found that the microstructure of almond yogurts was heterogeneous, containing aggregated protein particles, fat globules, fat and protein aggregates, and varying sizes and shapes of starch granules. A few dense clumps of fat and protein were also seen in the samples. Hydrophobic interactions due to protein denaturation and/or aggregation during processing may have caused these formations. Additionally, the presence of free fat globules in the matrix suggested that the fat may not have fully homogenized during production or may have partially coalesced during or after production. Conversely, yogurts made from cow's milk exhibited more homogeneous and protein‐based molecular network structures (Devnani et al. 2020).

### 3.8 Textural Features

Texture is an important characteristic that simulates chewing in the mouth (Huang et al. 2022). Despite being a good alternative to dairy yogurts due to their fats, proteins, and antioxidants, the inadequate textural properties of plant‐based yogurts limit their production and consumption. Studies on yogurts produced using plant‐based milks generally aim to improve the textural qualities of the products (Shi et al. 2020). Devnani et al. (2020) noted that stabilizers such as natural or modified starch, pectin, and gums, used in the formulation, affected the textural properties of commercial yogurt‐like products based on plant‐based milks. Hardness is defined as the maximum force that can occur at the gel's breaking point. The maximum negative force is taken as an indicator of the sample's cohesiveness; thus, the more negative the value, the stickier the sample (Angioloni and Collar 2009). Cohesiveness reflects the ability of the product to form a uniform structure. Adhesiveness represents the effort required for jaw movement due to stickiness in the mouth (Zhao et al. 2021). Table 6 presents the effect of using different ratios of PHP and storage time on the hardness, cohesiveness, adhesiveness, and gumminess values of yogurts. We observed that adding PHP in low amounts (0.125%) negatively impacted the yogurt's hardness. Similarly, a softer yogurt gel was obtained in the sample with 0.5% PHP added. On the first day of storage, the control sample exhibited the highest hardness value (41.16), with the closest result observed in the sample supplemented with 0.25% PHP (32.79). Adhesiveness showed a similar trend, with the control sample showing the highest value (188.13 g.s) and the sample supplemented with 0.25% PHP coming in second (150.53 g.s), respectively. The analysis measured the lowest adhesiveness values on the first day of storage and observed the highest values on the 21st day. Additionally, the trend in adhesiveness correlated with the results obtained from apparent viscosity measurements. Since higher hardness values indicate a tighter textural structure, the addition of *P. ovata* resulted in yogurts with a softer and creamier texture. The hardness values of all formulations showed an increase on the 7th day of storage, followed by a decrease on the 14th day, and then another increase on the 21st day. These changes were found to be statistically significant (*p* < 0.05). When comparing the adhesiveness values of all samples stored for 1 day, significant differences were observed (*p* < 0.05). Overall, it was observed that with increasing storage time, the increase in adhesiveness values of the samples was statistically significant (*p* < 0.05). As the proportion of PHP added to the yogurt samples increased, the cohesiveness values generally showed an increase. This increase created a statistically significant difference throughout the storage period (*p* < 0.05). The coconut milk yogurt sample with 0.25% PHP had the highest cohesiveness values on the first day of storage. This meant that the gel structure was stronger, which was supported by SEM images and sensory analysis results. Gumminess, which is an indicator of strong bonding in yogurt, is also important for its structural integrity. The coconut milk yogurt with 0.25% PHP had the highest gumminess value of 18.84 on the first day of storage. As the storage period increased, the gumminess values of the samples showed both increases and decreases, and these changes were statistically significant (*p* < 0.05). It is presumed that adding 0.25% PHP to the production of coconut milk‐based yogurts will increase the three‐dimensional network structure, leading to higher gumminess values. This result suggests that *P. ovata* can be considered a stabilizer in the production of plant‐based yogurts. These textural indices indicate that the gel‐like structure formed by coconut milk can be easily influenced by different proportions of *P. ovata* addition. Researchers found that adding *P. ovata* to low‐fat yogurt samples increased the gumminess values because it formed a strong polymer structure between the casein micelles and arabinoxylan chains (Ladjevardi et al. 2015). During a 21‐day storage period at 4°C, probiotic yogurts made with almond milk displayed fluctuations in hardness values ranging from 11.21 to 11.84. Adhesiveness values ranged from −7.09 to −6.27 (Yilmaz‐Ersan and Topcuoglu 2022). Almond milk‐based yogurts containing varying amounts of protein, fat, and sugar were found to have hardness values ranging from 4.69 g to 6.56 g, adhesiveness values ranging from 62.65 g.s to 75.40 g.s, and cohesiveness values ranging from 0.62 to 1.21. The highest hardness value (6.56 g) observed in yogurt‐like samples made from almond milk containing 7% fat was associated with the formulation's highest total solids content (15.15%) (Zhao et al. 2021). The hardness and adhesiveness values obtained in our study are notably higher compared to those reported by Zhao et al. (2021). One of the primary reasons for this could be the high dry matter and saturated fat content derived from coconut milk in our yogurt‐like products. Huang et al. (2022) reported that the addition of protein to plant‐based yogurts leads to the formation of a more durable gel network. Yogurts formulated with different proportions of soybean and quinoa exhibited hardness values ranging from 43.74 g to 52.41 g, adhesiveness values ranging from 0.89 to 0.93, and gumminess values ranging from 40.84 to 46.88. The increased cross‐linking of the gel network, resulting in a denser and firmer structure due to the higher protein and total solids content, could be the cause of the increase in hardness (Zhao et al. 2021).

**TABLE 6:** Texture profile analysis results of coconut milk‐based probiotic yogurts during 21 days of storage at 4°C.

PHP (%)	Storage (day)	Hardness	Cohesiveness	Adhesiveness	Gumminess
Control	1	41.16 ± 0.10^aC^	0.32 ± 0.03^cA^	188.13 ± 11.81^aD^	13.23 ± 1.21^bD^
	7	125.60 ± 0.30^aA^	0.21 ± 0.02^cC^	268.39 ± 22.66^aC^	26.37 ± 2.17^aA^
	14	75.55 ± 0.51^aB^	0.26 ± 0.01^cB^	314.43 ± 6.36^aB^	19.62 ± 0.74^aB^
	21	125.61 ± 2.71^aA^	0.13 ± 0.02^cD^	457.90 ± 92.44^aA^	16.38 ± 2.80^aC^
0.125	1	26.39 ± 1.27^cC^	0.43 ± 0.01^bA^	122.39 ± 0.75^dD^	11.26 ± 0.28^cA^
	7	29.73 ± 0.11^cB^	0.40 ± 0.01^bA^	140.12 ± 1.77^cC^	11.84 ± 0.28^cA^
	14	27.66 ± 3.93^bC^	0.32 ± 0.01^bB^	242.54 ± 14.60^bA^	8.92 ± 1.50^cB^
	21	31.23 ± 0.79^cA^	0.32 ± 0.02^aB^	195.95 ± 1.10^dB^	9.96 ± 0.79^bB^
0.25	1	32.79 ± 0.37^bB^	0.57 ± 0.02^aA^	150.53 ± 0.95^bD^	18.84 ± 0.37^aA^
	7	38.44 ± 0.88^bA^	0.41 ± 0.00^bB^	177.59 ± 0.79^bC^	15.79 ± 0.55^bB^
	14	21.97 ± 0.92^bC^	0.44 ± 0.02^aB^	197.84 ± 7.31^cB^	9.70 ± 0.95^cC^
	21	36.39 ± 1.23^cA^	0.24 ± 0.00^bC^	281.59 ± 17.30^bA^	8.76 ± 0.35^bC^
0.5	1	27.50 ± 1.47^cB^	0.53 ± 0.02^aA^	137.05 ± 0.19^cD^	14.74 ± 0.21^bB^
	7	28.75 ± 0.74^cB^	0.48 ± 0.01^aB^	171.61 ± 13.38^bC^	13.93 ± 0.51^cB^
	14	24.64 ± 2.07^bC^	0.46 ± 0.03^aB^	192.45 ± 9.28^cB^	11.20 ± 0.18^bC^
	21	57.81 ± 1.18^bA^	0.32 ± 0.05^aC^	260.76 ± 24.30^cA^	18.60 ± 2.55^aA^

*Note:* There are significant differences (*p* < 0.05) in the mean values of different capital letters (A–D: storage time at 4°C) in the product with the same formulation. The mean values of different lowercase letters (a–d: proportion of PHP added) on the same storage day are significantly different (*p* < 0.05).

### 3.9 Rheological Properties

**FIGURE 3:**
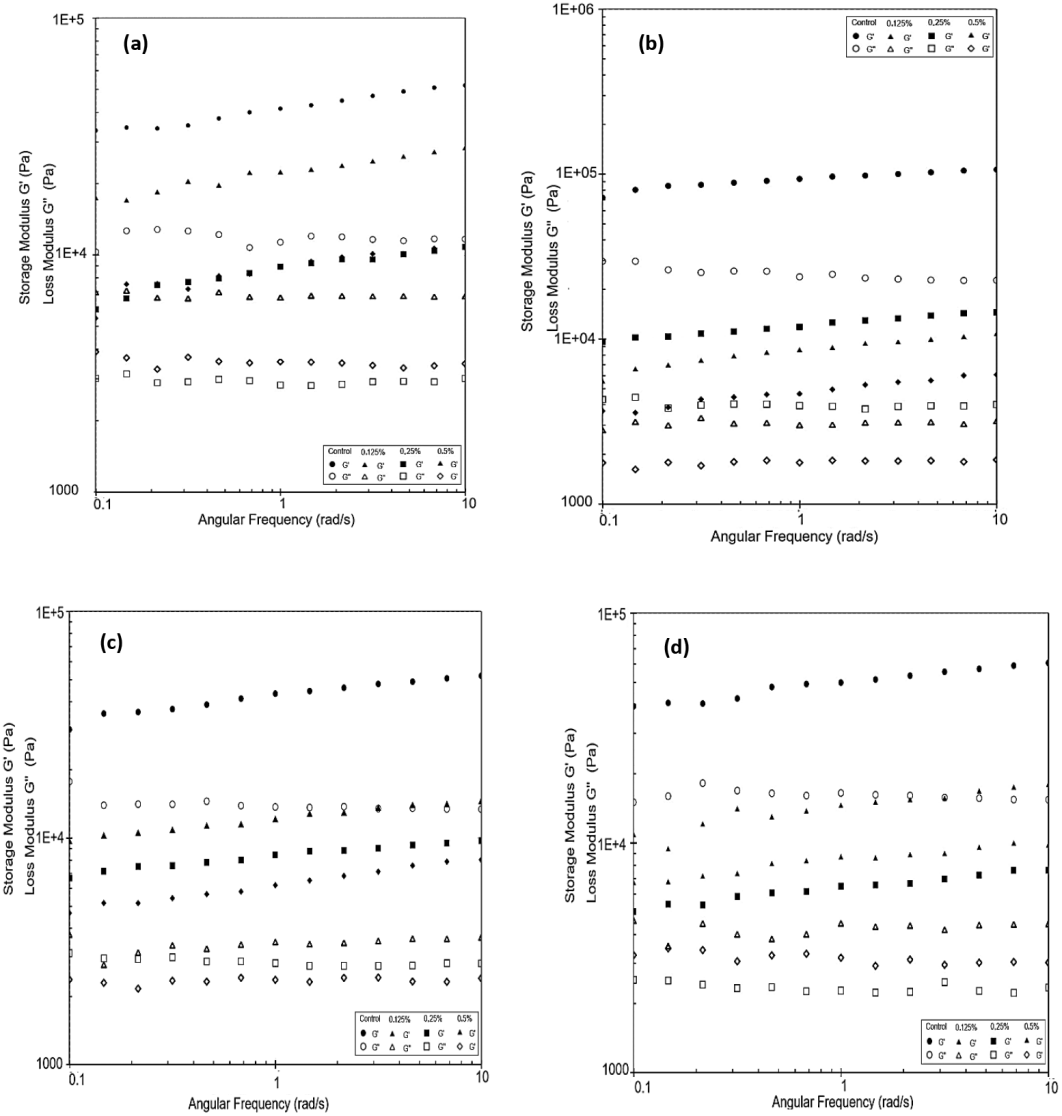
G′ and G′′ of probiotic yogurt products made from coconut milk measured on Day 1 (a), Day 7 (b), Day 14 (c), and Day 21 (d).

The addition of gelling agents and hydrocolloids such as agar, starch, gums, and/or pectin has a significant impact on the rheological properties of yogurts produced with plant‐based milks. The food industry commonly uses combinations of these additives to achieve the desired texture, either directly or through polysaccharide‐protein interactions (Grasso et al. 2020). Frequency sweeps are represented by two critical factors, storage (G′) and loss (G″) modulus (Hickisch et al. 2016). The storage modulus (G') expresses the elastic properties of acid gels and reflects their solid behavior (Zhao et al. 2021). The loss modulus, also known as the viscous modulus (G”), is an indicator of the fluidic character of the food system (Wu et al. 2009). In this study, dynamic rheological parameters, such as storage modulus (G') and loss modulus (G”), determined to investigate the internal structure of yogurt, as illustrated in Figure 3a–d. In coconut yogurts, a high G′ value indicates strong gel strength. As the amount of added PHP increases, the slight decrease in G′ may be related to a reduction in the gel strength of the gel matrix. Throughout the storage period, all coconut milk‐based yogurt samples exhibited the typical properties of a weak viscoelastic gel. G′ was consistently higher than G″ across the entire measurement range.

**FIGURE 4:**
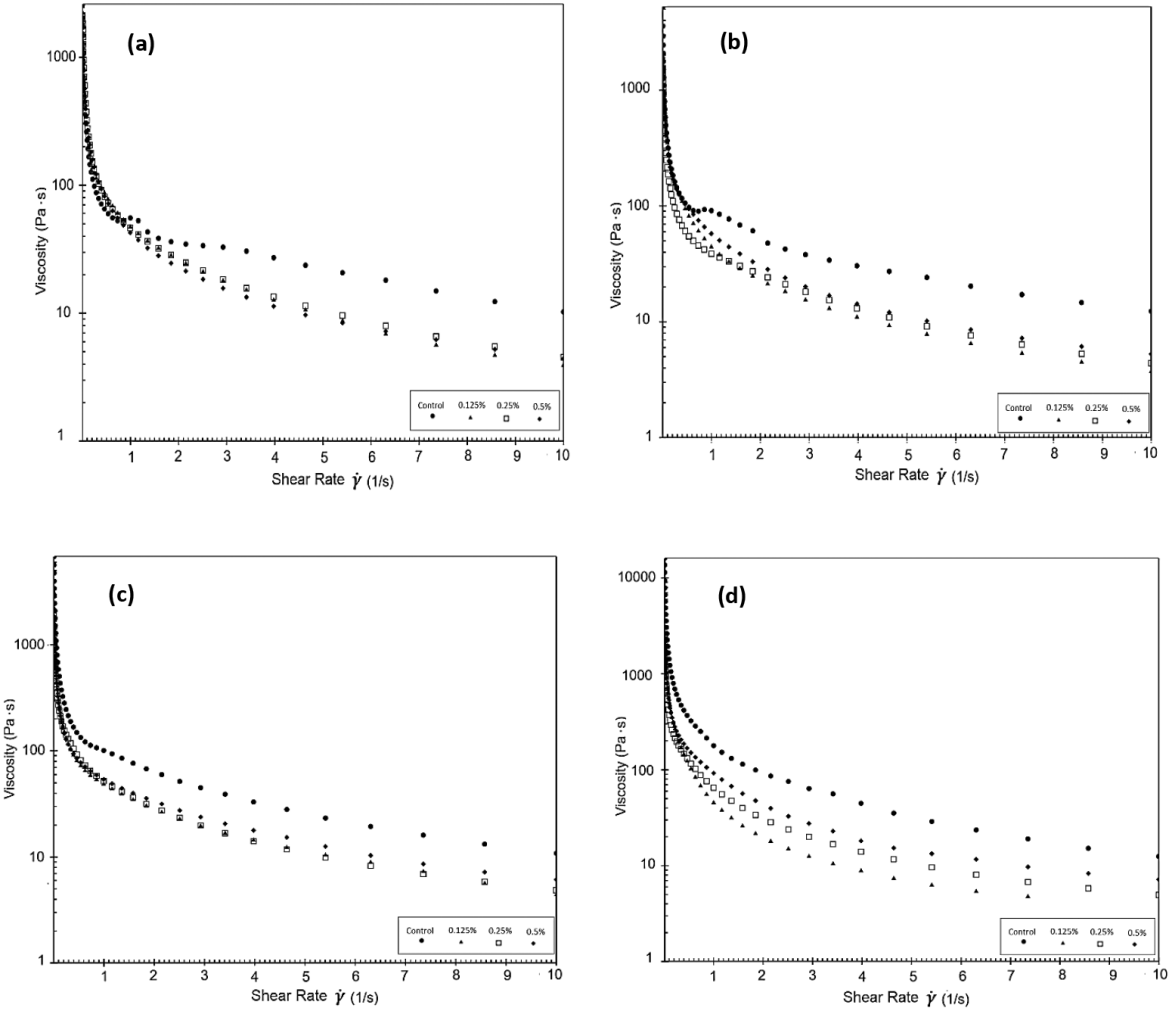
Apparent viscosity of probiotic yogurt products made from coconut milk measured on Day 1 (a), Day 7 (b), Day 14 (c), and Day 21 (d).

In a previous study (Pachekrepapol et al. 2021), the addition of tapioca starch to coconut milk‐based yogurts has been noted to increase G′, forming a gel‐like structure as the starch concentration increases. During a 14‐day storage period, it has been reported that G' is higher than G” for coconut milk‐based yogurts across the frequency range, and all samples typically exhibit a weak viscoelastic gel (G′>G″). As the concentration of tapioca starch added to the products increases, the G' values increase, with the control sample showing the lowest G' values. These findings are consistent with the results obtained in our study. During the fermentation stage of almond yogurts, there is a linear increase in G′ as the solid matter concentration (protein, fat, and sugar) increases (Zhao et al. 2021). Figure 4a–d display the viscosity values of the coconut milk‐based probiotic yogurts produced. As the shear rate increased, the apparent viscosity values of yogurt samples decreased. The decrease in viscosity during shearing indicates the shear‐thinning behavior of the samples. Table 7 presents the apparent viscosity values of the yogurt samples at 5.41 1/s. It was observed that the viscosity of the control sample was higher than that of the other samples and decreased during the storage period. The results showed that the viscosity of the yogurt samples increased in accordance with the rate of psyllium added to the samples, similar to the control sample. Conversely, the viscosity of the samples containing 0.25% and 0.5% PHP increased during the storage period. It is thought that the decrease in viscosity is caused by the weaker complexes that form between the polysaccharides in psyllium and the protein chains in coconut milk as the amount of powder added rises. PHP's high hydrophilic property led to the formation of a gel with a lower viscosity. It is believed that the number of bonds in the gel network and thus the gel strength are higher in yogurt produced with 0.5% PHP compared to yogurts with 0.125% and 0.25% PHP additions. On the 21st day of storage, the products with a 0.5% PHP addition were more viscous compared to those with a 0.25% addition. It is thought that as the storage time goes up, structural changes happen in the *P. ovata* inside the product. These changes cause the continuous phase to absorb water more quickly and the gel network to get stronger, which makes the product thicker. The rheological study of plant‐based yogurt made from quinoa and soybeans showed that as the shear rate went up, the apparent viscosity went down. This is called non‐Newtonian shear‐thinning behavior. All yogurt samples exhibited weak viscoelastic gel behavior (G′>G″) (Huang et al. 2022). The viscosity graph in a study examining the rheological properties of commercial plant‐based yogurts revealed that all samples exhibited shear‐thinning behavior. Almond yogurts exhibited a greater magnitude of shear thinning than soy yogurts. This behavior was attributed to the absence of an organized protein‐based network structure found in soy and dairy yogurts and almond yogurts (Devnani et al. 2020). Almond yogurt samples exhibited shear thinning behavior, with viscosity decreasing as shear rate increased. The increase in protein content resulted in higher viscosity values. The most effective factor in the decrease and increase of viscosity was the interaction between protein and fat in the formulation. Zhao et al. (2021) highlighted the impact of fat droplets coating the proteins on the gel network. The higher viscosity of almond milk‐based yogurts with increased total solids content has been associated with a more rigid network structure (Zhao et al. 2021). Studies by Marafon et al. (2011) and Geremias‐Andrade et al. (2016) have also reported data supporting the increase in viscosity due to the increase in total solids content. For instance, when the total solids content of milk was increased from 12.32% to 15.68%, the apparent viscosity of the yogurt broken at 1 s^−1^ shear rate increased from 14.72 Pa·s to 22.72 Pa·s (Wu et al. 2009). In a commercial coconut yogurt formulation containing water, coconut cream (20%), modified corn starch, dextrose, salt, thickener (pectin), colorant (carotene), calcium phosphate, and yogurt cultures ( *S. thermophilus* and *Lb. bulgaricus*), the apparent viscosity at 200 s^−1^ was found to be 0.75 Pa·s (Grasso et al. 2020). In a study where a combination of 0.6% polymerized whey protein, 0.3% pectin, and 0.05% xanthan gum was used as a gelling agent in almond milk‐based yogurts, the viscosity values were 47.37 mPa·s in the control sample and 41.22 mPa·s in the sample with the gelling agents. Shi et al. (2020) observed changes in viscosity during a 4‐week storage period. The increases and decreases in viscosity values observed are consistent with the findings reported by Shi et al. (2020). Compared to the apparent viscosity of cow milk yogurt (~2000–2400 mPa.s at 50 s^−1^) reported by Lee and Lucey (2006), the apparent viscosity of coconut yogurt obtained in our study was significantly lower. This indicates that coconut yogurt gels are characterized by a weaker gel network compared to yogurt gels produced from cow milk. *Plantago ovata* , with its high molecular weight and gel‐forming potential, has increased the hardness and viscosity of low‐fat yogurts. This could be attributed to the presence of acidic arabinoxylan with a 1 → 4 bond in the xylan backbone of the alkaline‐extractable gel‐forming fraction, which has a large average hydrodynamic diameter (> 100 nm). It has also been noted that the presence of highly branched acidic arabinoxylan in the structure of psyllium could hinder the formation of stronger gels by preventing molecules from associating with each other when added in excess to the formulation. An example provided by Ladjevardi et al. (2015) demonstrated that a yogurt sample containing 0.12% psyllium husk gum and 0.83% fat yielded the highest hardness (0.222 N) and viscosity (9.0 Pa·s) values.

**TABLE 7:** Apparent viscosities of coconut milk‐based probiotic yogurts at 5.41 1/s during 21 days of storage at 4°C.

PHP (%)	Storage (day)	Viscosity (Pa.s)
Control	1	23.564 ± 0.54^aA^
	7	22.083 ± 0.51^aB^
	14	17.660 ± 0.38^aC^
	21	15.955 ± 0.37^aD^
0.125	1	9.119 ± 0.30^cA^
	7	7.973 ± 0.27^dB^
	14	7.887 ± 0.27^dB^
	21	6.393 ± 0.26^dC^
0.25	1	9.184 ± 0.31^cB^
	7	8.977 ± 0.28^cC^
	14	10.391 ± 0.32^cA^
	21	9.713 ± 0.31^cB^
0.5	1	10.713 ± 0.33^bC^
	7	10.044 ± 0.32^bC^
	14	14.329 ± 0.35^bA^
	21	13.089 ± 0.34^bB^

*Note:* There are significant differences (*p* < 0.05) in the mean values of different capital letters (A–D: storage time at 4°C) in the product with the same formulation. The mean values of different lowercase letters (a–d: proportion of PHP added) on the same storage day are significantly different (*p* < 0.05).

### 3.10 Sensory Evaluation

**FIGURE 5:**
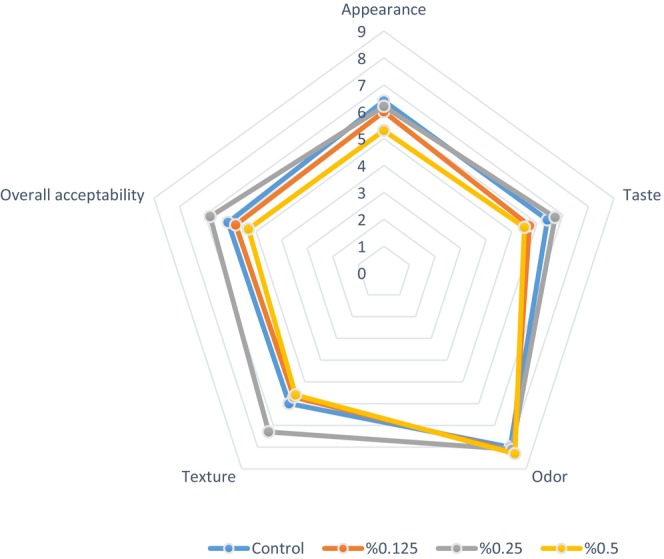
Sensory evaluation of coconut milk‐based probiotic yogurts performed after 24 h of storage at 4°C.

Sensory characteristics have a significant impact on food acceptance. The characteristic taste and texture of yogurt are functions of milk components, including milk fat, protein, and carbohydrates, as well as the presence and metabolism of LAB during fermentation (Chen et al. 2017). Figure 5 displays a spider web diagram representation of the sensory analysis results of plant‐based probiotic yogurts stored at 4°C for 24 h. Average sensory scores for all probiotic yogurt samples fell within an acceptable range (4–8). Yogurt samples with 0.25% PHP received significantly higher scores in terms of taste, texture, and overall acceptability (*p* < 0.05). This finding is consistent with the results obtained from microstructural and textural analyses. The addition of 0.5% PHP resulted in the lowest score (5.3) for the appearance attribute. It was considered that this score could correspond to the color parameters measured with a colorimeter in the color analysis results. We found the difference in odor scores among yogurts with different ratios of PHP to be statistically insignificant (*p* > 0.05). This result is supported by the fact that PHP has no effect on the odor. Adding 1.0%, 1.5%, and 2.0% tapioca starch to coconut milk resulted in yogurt‐like products that received higher scores in overall acceptability, texture, and flavor compared to the control (Pachekrepapol et al. 2021). Increasing the proportion of psyllium gel added to the liquid Kashk content from 0.1% to 0.5% led to a significant increase in taste and overall acceptability scores, while adding psyllium gel at 0.75% reduced these sensory attributes (Amini et al. 2018). According to the sensory evaluation results in the study conducted by Choobari et al. (2021), there was no significant difference in taste between control samples that had *P. ovata* Forsk seed mucilage and those that did not. Probiotic yogurt containing 1% *P. ovata* demonstrated the highest acceptability in terms of appearance and texture. The positive contributions of *P. ovata* addition to sensory parameters are consistent with the findings obtained in our study.

### 3.11 Principle Component (PC) and Hierarchical Clustering Analyses

**FIGURE 6:**
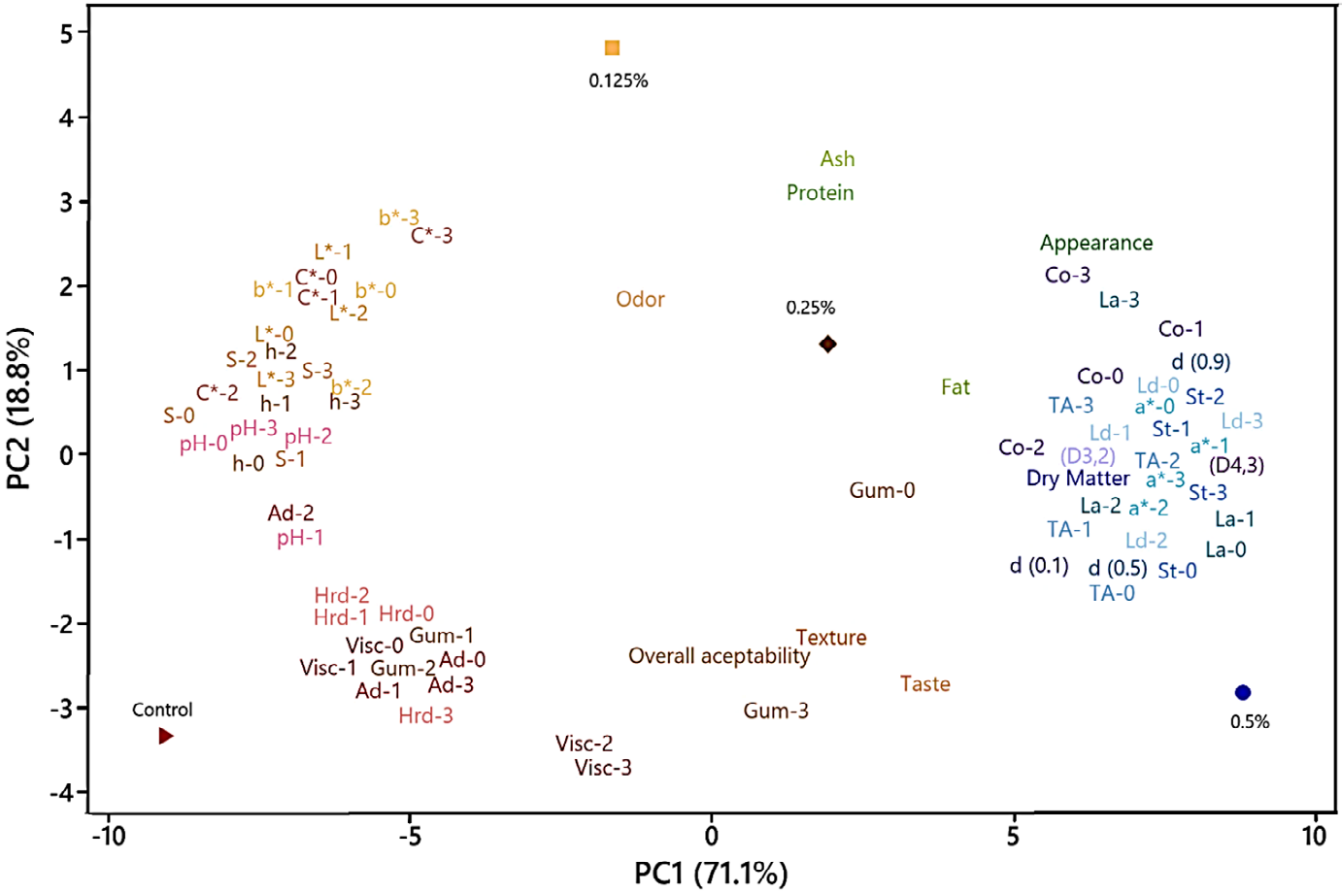
Loading plots and score plots of the first two principal components (PC1 and PC2) of PCA analysis describing the variation between yogurt samples (Gum: Gumminess; Co: Cohesiveness; La: *Lb. acidophilus*; Ld: *Lb. delbrueckii* subsp. *bulgaricus*; St: *S. thermophilus*, TA: Titratable acidity; Visc: Viscosity; Ad: Adhesiveness; Hrd: Hardness and the numerals “0,” “1,” “2,” and “3” next to the expressions indicate the respective storage periods).

**FIGURE 7:**
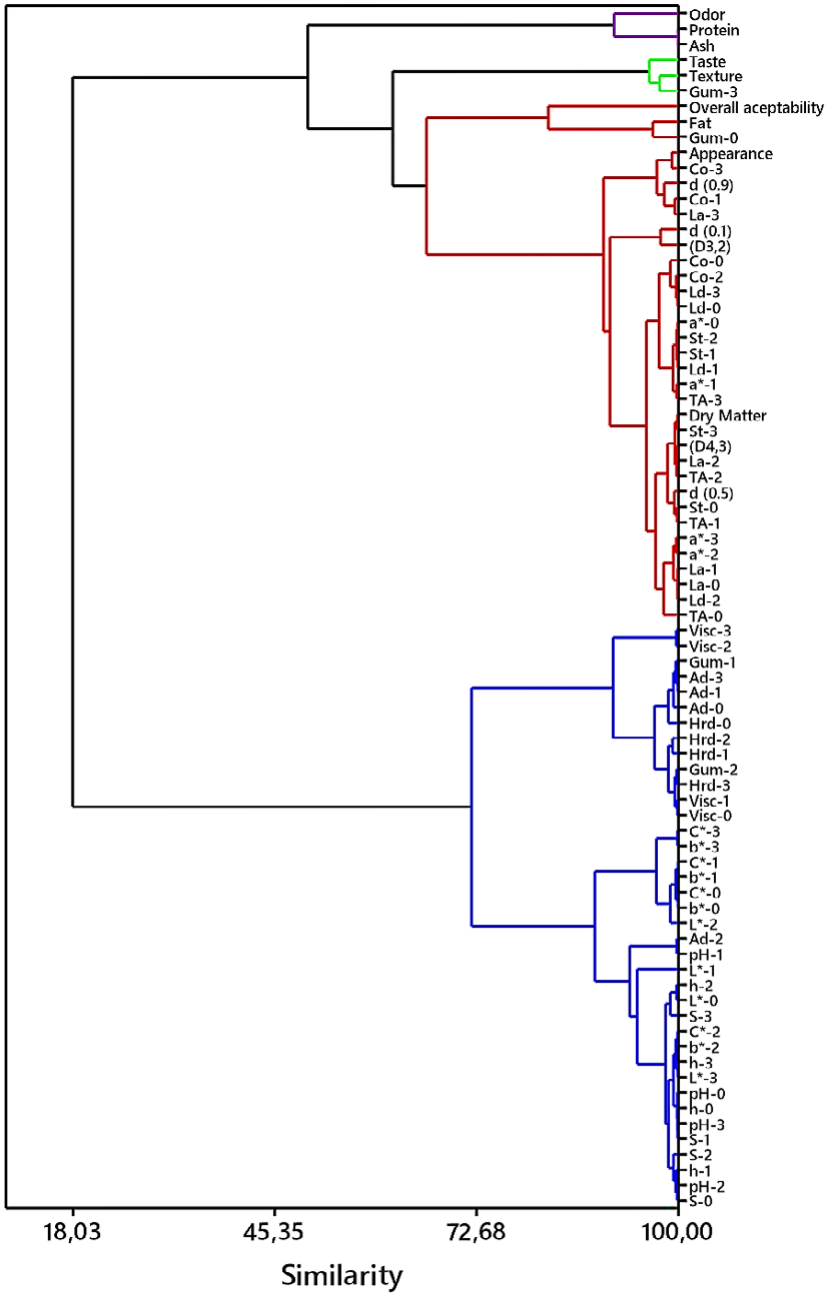
The dendrogram of variable clustering. Variables that are in the same color were grouped together because they are similar to each other. The groups joined with the next group until just one cluster was formed at the final step (Gum: Gumminess; Co: Cohesiveness; La: *Lb. acidophilus*; Ld: *Lb. delbrueckii* subsp. *bulgaricus*; St: *S. thermophilus*, TA: Titratable acidity; Visc: Viscosity; Ad: Adhesiveness; Hrd: Hardness and the numerals “0,” “1,” “2,” and “3” next to the expressions indicate the respective storage periods).

A hierarchical cluster (HCA) and principal component (PCA) analyses were performed on the data to identify discrepancies between the yogurt samples and to determine the influence of rheological, microstructural, microbiological, physicochemical, and sensorial attributes on these variations. The results of the analyses are presented in Figures 6 and 7. Three principal components (PC1, PC2, PC3) were identified which explained 71.1%, 18.8%, and 10.1% of the variation in protein, ash, dry matter, pH, acidity, syneresis, viscosity, color parameters, textural, sensory, and microbiological properties of yogurt samples (Table 8). The contribution of PC1 and PC2 to the total variation was 89.9%. The control sample and the samples containing 0.5%, 0.25%, and 0.125% of PHP were located in quadrants III, IV, I, and II, respectively. Odor, hardness, gumminess, adhesiveness, viscosity, pH, *h*, L*, and syneresis were highly correlated with PC1, and the control sample was discriminated from other samples by these properties. The control sample was distinguished from the other samples based on its adhesiveness, gumminess, viscosity, hardness, pH, L*, and b* values. Conversely, the sample containing 0.5% PHP was differentiated from the others based on its *Lb. delbrueckii* subsp. *bulgaricus, S. thermophilus, Lb. acidophilus*, TA, cohesiveness, dry matter, and particle size and appearance properties. According to the results of cluster analysis, two main clusters were formed (Figure 7). The first main cluster consisted of two sub‐clusters, and viscosity, adhesiveness, hardness, C*, b*, L*, syneresis, and pH were effective in this clustering. The first main cluster represents the control sample. The second main cluster was further divided into three sub‐clusters. One sub‐cluster was divided according to odor, protein, and ash characteristics, and represents the sample containing 0.125% PHP. The second sub‐cluster was divided according to texture, taste, and gumminess characteristics, representing the sample containing 0.25% PHP. The third sub‐cluster was divided according to overall acceptability, fat, appearance, dry matter, cohesiveness, *Lb. delbrueckii* subsp. *bulgaricus, S. thermophilus, Lb. acidophilus*, a*, TA, and particle size characteristics, representing the 0.5% sample. The cluster analysis results demonstrate a notable similarity to the PCA. Principal component analysis and hierarchical cluster analyses are alternative statistical methods to reveal the differences between groups considering all quality parameters. These two methods were used to express the relationship between physillium utilization rate and quality parameters in coconut milk yogurt production and to determine the degree of influence on the variation of quality parameters.

**TABLE 8:** Eigenvalue, percentage of variance, and cumulative variance for the components.

Components	Eigenvalue	% of variance	Cumulative (%)
PC1	55.487	71.1	71.1
PC2	14.647	18.8	89.9
PC3	7.866	10.1	100

## 4 Conclusion

In this study, the effects of incorporating different proportions of PHP into the formulation of plant‐based yogurt produced by adding probiotic culture (*Lb. delbrueckii* subsp. *bulgaricus, S. thermophilus*, and *Lb. acidophilus*) to coconut milk were evaluated. The addition of PHP, a source of hydrocolloid production, altered the syneresis, viscosity, rheological, and textural properties of plant‐based yogurt samples. The results of a microbiological count revealed that PHP had a positive effect on the growth and metabolic activity of LAB. The coconut milk‐based yogurts with PHP had a 14‐day shelf life that was more suitable in terms of probiotic viability. When the microstructures of coconut milk yogurts visualized by SEM were evaluated, it was determined that the most homogeneous gel structure was in the products to which 0.25% PHP was added, and it was determined that this rate improved the textural and rheological properties. In addition, PHP was not found to have any negative effect on the sensory properties of yogurt samples, and yogurt containing 0.25% PHP had the highest scores in taste, texture, and overall acceptability parameters. In future studies, different plant‐based milks can be used as an alternative to coconut milk, and product optimizations can be made by adding PHP as a stabilizer to the formulation in the production of plant‐based yogurts, and the shelf life of the products can be examined. In this way, it is thought that it will contribute to increasing the potential of functional products to become a viable production process for consumers who have health problems such as lactose intolerance and cow's milk protein allergy.

## Author Contributions

**Ayca Gülhan:** conceptualization (equal), formal analysis (equal), funding acquisition (equal), investigation (equal), methodology (equal), project administration (lead), resources (equal), software (equal), writing – original draft (equal), writing – review and editing (equal). **Hacer Çoklar:** formal analysis (equal), investigation (equal), methodology (equal), supervision (equal), writing – original draft (equal), writing – review and editing (equal). **Mehmet Akbulut:** supervision (equal), writing – review and editing (equal).

## Ethics Statement

The authors have nothing to report.

## Consent

Written informed consent was obtained from all study participants.

## Conflicts of Interest

The authors declare no conflicts of interest.

## Data Availability

The data that support the findings of this study are available on request from the corresponding author.

## References

[fsn370085-bib-0001] Abdelmoneim, A. H. , A. M. Sherif , and K. A. Sameh . 2016. “Rheological Properties of Yoghurt Manufactured by Using Different Types of Hydrocolloids.” Austin Journal of Nutrition and Food Sciences 4, no. 2: 1082.

[fsn370085-bib-0002] AL‐Ogaidi, A. A. A. , and A. A. Alshaikh Daher . 2019. “The Study of Some Chemical Properties of *Plantago ovata* Seeds and Its Utilization as Synbiotics in Fermented Milk.” Plant Archives 19, no. 2: 1824–1829.

[fsn370085-bib-0003] Amini, S. , S. Yousefi , and A. A. Moghari . 2018. “Development and Quality Characterization of Liquid Kashk by Incorporating Psyllium (*Plantago ovata* Forsk) Hydrocolloid Gel.” Journal of Food Measurement and Characterization 12: 1669–1677. 10.1007/s11694-018-9782-8.

[fsn370085-bib-0004] Angioloni, A. , and C. Collar . 2009. “Small and Large Deformation Viscoelastic Behaviour of Selected Fibre Blends With Gelling Properties.” Food Hydrocolloids 23: 742–748. 10.1016/j.foodhyd.2008.04.005.

[fsn370085-bib-0005] AOAC . 2005. Official Methods of Analysis, Association of OfficialAnalytical Chemistry. 16th ed. Association of Official Analysis Chemists International.

[fsn370085-bib-0006] Ashraf, R. , and S. C. Smith . 2015. “Selective Enumeration of Dairy Based Strains of Probiotic and Lactic Acid Bacteria.” International Food Research Journal 22, no. 6: 2576–2586.

[fsn370085-bib-0007] Aydoğan‐Coşkun, B. , M. Ercan , M. Akbulut , et al. 2022. “Influence of Grafting on Fatty Acid Profile and Some Physicochemical Properties of Watermelon Seed and Seed Oil.” Grasas y Aceites 73, no. 3: e475. 10.3989/gya.0784211.

[fsn370085-bib-0008] Bernat, N. , M. Chafera , A. Chiralt , and C. Gonzalez‐Martınez . 2015. “Probiotic Fermented Almond “Milk” as an Alternative to Cow‐Milk Yoghurt.” International Journal of Food Studies 4: 201–211. 10.7455/ijfs/4.2.2015.a8.

[fsn370085-bib-0009] Bilican, I. 2023. “Preparation and Properties of Novel Mucilage Composite Films Reinforced With Polydimethylsiloxane.” Macromolecular Materials and Engineering 309, no. 3: 2300317. 10.1002/mame.202300317.

[fsn370085-bib-0010] Boeck, T. , A. W. Sahin , E. Zannini , and E. K. Arendt . 2021. “Nutritional Properties and Health Aspects of Pulses and Their Use in Plant‐Based Yogurt Alternatives.” Comprehensive Reviews in Food Science and Food Safety 20: 3858–3880. 10.1111/1541-4337.12778. 34125502

[fsn370085-bib-0011] Bruno, L. M. , J. R. Lima , N. J. Wurlitzer , and T. C. Rodrigues . 2020. “Non‐Dairy Cashew Nut Milk as a Matrix to Deliver Probiotic Bacteria.” Food Science and Technology 40, no. 3: 604–607. 10.1590/fst.14219.

[fsn370085-bib-0012] Chen, C. , S. Zhao , G. Hao , H. Yu , H. Tian , and G. Zhao . 2017. “Role of Lactic Acid Bacteria on the Yogurt Flavour: A Review.” International Journal of Food Properties 20, no. S1: 316–330. 10.1080/10942912.2017.1295988.

[fsn370085-bib-0013] Choobari, S. Z. M. , A. A. Sari , and A. D. Garmakhany . 2021. “Effect of *Plantago ovata* Forsk Seed Mucilage on Survivability of *Lactobacillus acidophilus*, Physicochemical and Sensory Attributes of Produced Low‐Fat Set Yoghurt.” Food Science & Nutrition 9: 1040–1049. 10.1002/fsn3.2074. 33598187 PMC7866596

[fsn370085-bib-0014] Coklar, H. , and M. Akbulut . 2010. “Effect on Phenolics, HMF and Some Physico‐Chemical Properties of Apple Juice Concentrate of Activated Carbon Applied at the Different Temperatures.” Journal of Food Process Engineering 33, no. 2: 370–383. 10.1111/j.1745-4530.2008.00280.x.

[fsn370085-bib-0015] Craig, W. J. , and C. J. Brothers . 2021. “Nutritional Content and Health Profile of Non‐Dairy Plant‐Based Yogurt Alternatives.” Nutrients 13, no. 11: 4069. 10.3390/nu13114069. 34836324 PMC8619131

[fsn370085-bib-0016] Dawane, D. D. , R. C. Ranveer , and K. D. Nagargoje . 2010. “Utilization of Tender Coconut (*Cocus nucifera* L.) Milk in the Preparation of Pudding.” Food Science Research Journal 1, no. 2: 111–115.

[fsn370085-bib-0017] Devnani, B. , L. Ong , S. Kentish , and S. Gras . 2020. “Heat Induced Denaturation, Aggregation and Gelation of Almond Proteins in Skim and Full Fat Almond Milk.” Food Chemistry 325: 126901. 10.1016/j.foodchem.2020.126901. 32387956

[fsn370085-bib-1001] Devnani, B. , L. Ong , S. E. Kentish , P. J. Scales , and S. L. Gras . 2022. “Physicochemical and Rheological Properties of Commercial Almond‐based Yoghurt Alternatives to Dairy and Soy Yoghurts.” Future Foods 6: 100185. 10.1016/j.fufo.2022.100185.

[fsn370085-bib-0018] Fan, X. , X. Li , L. Du , et al. 2022. “The Effect of Natural Plant‐Based Homogenates as Additives on the Quality of Yogurt: A Review.” Food Bioscience 49: 101953. 10.1016/j.fbio.2022.101953.

[fsn370085-bib-0019] Firooz, F. , A. Nemati , and N. Abbasgholizadeh . 2019. “The Effect Mucilages of *Plantago ovata* and *Salvia macrosiphon* as a Replacement of Fat on Sensory, Microbial and Durability of Low‐Fat Stirred Yoghurt Probiotics.” Journal of Health 10, no. 1: 98–108.

[fsn370085-bib-0020] Geremias‐Andrade, I. M. , N. P. B. G. Souki , I. C. F. Moraes , and S. C. Pinho . 2016. “Rheology of Emulsion‐Filled Gels Applied to the Development of Food Materials.” Gels 2, no. 22: 1–18. 10.3390/gels2030022. PMC6318578 30674153

[fsn370085-bib-0021] Grasso, N. , L. Alonso‐Miravalles , and J. A. O'Mahony . 2020. “Composition, Physicochemical and Sensorial Properties of Commercial Plant‐Based Yogurts.” Food 9: 252. 10.3390/foods9030252. PMC7142433 32110978

[fsn370085-bib-0022] Gülhan, A. , H. Çoklar , and M. Akbulut . 2023. “The Usability of Crab Apple (*Malus floribunda*) Anthocyanins as a Natural Colorant in Apple Marmalade.” Brazilian Archives of Biology and Technology 66: e23220660. 10.1590/1678-4324-2023220660.

[fsn370085-bib-0023] Hickisch, A. , R. Beer , R. F. Vogel , and S. Toelstede . 2016. “Influence of Lupin‐Based Milk Alternative Heat Treatment and Exopolysaccharide‐Producing Lactic Acid Bacteria on the Physical Characteristics of Lupin‐Based Yogurt Alternatives.” Food Research International 84: 180–188. 10.1016/j.foodres.2016.03.037. 28460988

[fsn370085-bib-0024] Huang, K. , Y. Liu , Y. Zhang , et al. 2022. “Formulation of Plant‐Based Yoghurt From Soybean and Quinoa and Evaluation of Physicochemical, Rheological, Sensory and Functional Properties.” Food Bioscience 49: 101831. 10.1016/j.fbio.2022.101831.

[fsn370085-bib-0025] IDF . 2013. “TS ISO 9622:2015. Milk and Liquid Milk Products – Guidelines for the Application of Mid‐Infrared Spectrometry No: 141.”

[fsn370085-bib-0026] ISO . 2020. “13320; Particle Size Analysis‐Laser Diffraction Methods.” ISO.

[fsn370085-bib-0027] Karimidastjerd, A. , Z. Gulsunoglu‐Konuskan , E. Olum , and O. S. Toker . 2024. “Evaluation of Rheological, Textural, and Sensory Characteristics of Optimized Vegan Rice Puddings Prepared by Various Plant‐Based Milks.” Food Science & Nutrition 12: 1779–1791. 10.1002/fsn3.3872. 38455179 PMC10916541

[fsn370085-bib-0028] Ladjevardi, Z. S. , S. M. T. Gharibzahedi , and M. Mousavi . 2015. “Development of a Stable Low‐Fat Yogurt Gel Using Functionality of Psyllium (*Plantago ovata* Forsk) Husk Gum.” Carbohydrate Polymers 125: 272–280. 10.1016/j.carbpol.2015.02.051. 25857984

[fsn370085-bib-0029] Lee, W. J. , and J. A. Lucey . 2006. “Impact of Gelation Conditions and Structural Breakdown on the Physical and Sensory Properties of Stirred Yogurts.” Journal of Dairy Science 89: 2374–2385. 10.3168/jds.S0022-0302(06)72310-4. 16772553

[fsn370085-bib-0030] Madgulkar, A. R. , M. R. P. Rao , and D. Warrier . 2014. “Characterization of Psyllium (*Plantago ovata*) Polysaccharide and Its Uses.” In Polysaccharides, edited by K. Ramawat and J. M. Mérillon . Springer.

[fsn370085-bib-0031] Marafon, A. P. , A. Sumi , M. R. Alcantara , A. Y. Tamime , and M. de Nogueira Oliveira . 2011. “Optimization of the Rheological Properties of Probiotic Yoghurts Supplemented With Milk Proteins.” LWT‐Food Science and Technology 44, no. 2: 511–519. 10.1016/j.lwt.2010.09.005.

[fsn370085-bib-0032] Naibaho, J. , N. Butula , E. Jonuzi , M. Korzeniowska , G. Chodaczek , and B. Yang . 2022. “The Roles of Brewers' Spent Grain Derivatives in Coconut‐Based Yogurt‐Alternatives: Microstructural Characteristic and the Evaluation of Physico‐Chemical Properties During the Storage.” Current Research in Food Science 5: 1195–1204. 10.1016/j.crfs.2022.07.011. 35992631 PMC9382424

[fsn370085-bib-0033] Nazario Franco, E. A. , A. Sanches‐Silva , R. Ribeiro‐Santos , and N. de Ramos Melo . 2020. “Psyllium (*Plantago ovata* Forsk): From Evidence of Health Benefits to Its Food Application.” Trends in Food Science and Technology 96: 166–175. 10.1016/j.tifs.2019.12.006.

[fsn370085-bib-0034] Pachekrepapol, U. , Y. Kokhuenkhan , and J. Ongsawat . 2021. “Formulation of Yogurt‐Like Product From Coconut Milk and Evaluation of Physicochemical, Rheological, and Sensory Properties.” International Journal of Gastronomy and Food Science 25: 100393. 10.1016/j.ijgfs.2021.100393.

[fsn370085-bib-0035] Pandey, A. , S. S. Koruri , R. Chowdhury , and P. Bhattacharya . 2016. “Prebiotic Influence of *Plantago ovata* on Free and Microencapsulated *L. casei*‐Growth Kinetics, Antimicrobial Activity and Microcapsules Stability.” International Journal of Pharmacy and Pharmaceutical Sciences 8, no. 8: 89–97.

[fsn370085-bib-0036] Rasika, D. M. D. , J. K. Vidanarachchi , R. S. Rocha , et al. 2021. “Plant‐Based Milk Substitutes as Emerging Probiotic Carriers.” Current Opinion in Food Science 38: 8–20. 10.1016/j.cofs.2020.10.025.

[fsn370085-bib-0037] Shi, H. , J. Kraft , and M. Guo . 2020. “Physicochemical and Microstructural Properties and Probiotic Survivability of Symbiotic Almond Yogurt Alternative Using Polymerized Whey Protein as a Gelation Agent.” Journal of Food Science 85, no. 10: 3450–3458. 10.1111/1750-3841.15431. 32901954

[fsn370085-bib-0038] Shori, A. B. , G. S. Aljohani , A. J. Al‐zahrani , O. S. Al‐sulbi , and A. S. Baba . 2022. “Viability of Probiotics and Antioxidant Activity of Cashew Milk‐Based Yogurt Fermented With Selected Strains of Probiotic *Lactobacillus* spp.” LWT‐Food Science and Technology 153: 112482. 10.1016/j.lwt.2021.112482.

[fsn370085-bib-0039] Siefarth, C. , T. B. T. Tran , P. Mittermaier , T. Pfeiffer , and A. Buettner . 2014. “Effect of Radio Frequency Heating on Yoghurt, I: Technological Applicability, Shelf‐Life and Sensorial Quality.” Food 3: 318–335. 10.3390/foods3020318. PMC5302359 28234322

[fsn370085-bib-0040] Tulashie, S. K. , J. Amenakpor , S. Atisey , R. Odai , and E. E. A. Akpari . 2022. “Production of Coconut Milk: A Sustainable Alternative Plant‐Based Milk.” Case Studies in Chemical and Environmental Engineering 6: 100206. 10.1016/j.cscee.2022.100206.

[fsn370085-bib-0041] Wang, S. , V. Chelikani , and L. Serventi . 2018. “Evaluation of Chickpea as Alternative to Soy in Plant‐Based Beverages, Fresh and Fermented.” LWT‐Food Science and Technology 97: 570–575. 10.1016/j.lwt.2018.07.067.

[fsn370085-bib-0042] Wu, S. , D. Li , S. J. Li , et al. 2009. “Effects of Incubation Temperature, Starter Culture Level and Total Solids Content on the Rheological Properties of Yogurt.” International Journal of Food Engineering 5, no. 2: 1–17. 10.2202/1556-3758.1436.

[fsn370085-bib-0043] Yilmaz‐Ersan, L. , and E. Topcuoglu . 2022. “Evaluation of Instrumental and Sensory Measurements Using Multivariate Analysis in Probiotic Yogurt Enriched With Almond Milk.” Journal of Food Science and Technology 59, no. 1: 133–143. 10.1007/s13197-021-04994-w. 33583953 PMC7868674

[fsn370085-bib-0044] Zhao, J. , B. Bhandari , C. Gaiani , and S. Prakash . 2021. “Physicochemical and Microstructural Properties of Fermentation‐Induced Almond Emulsion‐Filled Gels With Varying Concentrations of Protein, Fat and Sugar Contents.” Current Research in Food Science 4: 577–587. 10.1016/j.crfs.2021.08.007. 34485926 PMC8405962

